# Body-Weight Fluctuation Was Associated With Increased Risk for Cardiovascular Disease, All-Cause and Cardiovascular Mortality: A Systematic Review and Meta-Analysis

**DOI:** 10.3389/fendo.2019.00728

**Published:** 2019-11-08

**Authors:** Huajie Zou, Ping Yin, Liegang Liu, Wenhua Liu, Zeqing Zhang, Yan Yang, Wenjun Li, Qunchuan Zong, Xuefeng Yu

**Affiliations:** ^1^Division of Endocrinology, Department of Internal Medicine, Tongji Hospital, Tongji Medical College, Huazhong University of Science and Technology, Wuhan, China; ^2^Department of Epidemiology and Biostatistics, School of Public Health, Tongji Medical College, Huazhong University of Science and Technology, Wuhan, China; ^3^Hubei Key Laboratory of Food Nutrition and Safety, Department of Nutrition and Food Hygiene, Tongji Medical College, Huazhong University of Science and Technology, Wuhan, China; ^4^Ministry of Education Key Lab of Environment and Health, School of Public Health, Tongji Medical College, Wuhan, China; ^5^Medical Translation Center, Tongji Medical College, Tongji Hospital, Huazhong University of Science and Technology, Wuhan, China; ^6^Computer Center, Tongji Hospital, Tongji Medical College, Huazhong University of Science and Technology, Wuhan, China; ^7^Department of Orthopedics, The Affiliated Hospital of Qinghai University, Medical College of Qinghai University, Xining, China

**Keywords:** body-weight fluctuation, weight cycling, mortality, CVD, meta-analysis

## Abstract

**Background:** The aim of this study was to evaluate associations between body-weight fluctuation and risk of mortality and cardiovascular diseases (CVD).

**Methods:** PubMed, EMBASE databases and Cochrane Library were searched for cohort studies published up to May 20, 2019, reporting on associations of body-weight fluctuation and mortality from all causes, CVD and cancer, as well as morbidity of CVD and hypertension. Summary relative risks (RRs) were estimated using a random-effects model.

**Results:** Twenty-five eligible publications from 23 studies with 441,199 participants were included. Body-weight fluctuation was associated with increased risk for all-cause mortality (RR, 1.41; 95% confidence interval (CI): 1.27–1.57), CVD mortality (RR, 1.36; 95% CI 1.22–1.52), and morbidity of CVD (RR, 1.49, 95% CI 1.26–1.76) and hypertension (RR, 1.35, 95% CI 1.14–1.61). However, there was no significant association between weight fluctuation and cancer mortality (RR, 1.01; 95% CI 0.90–1.13). No evidence of publication bias was observed (all *P* > 0.05) except for studies on all-cause mortality (Egger's test, *P* = 0.001; Begg's test, *P* = 0.014).

**Conclusions:** Body-weight fluctuation was associated with higher mortality due to all causes and CVD and a higher morbidity of CVD and hypertension.

## Introduction

Body-weight fluctuation refers to the repeated loss and regain of weight within a specific period (1, 2). Currently, no single definition or measurement is endorsed for weight fluctuation (1). Weight fluctuation is a common, partly due to the high prevalence of weight regain after weight loss by dieting or exercising in the individuals with overweight, obesity or even with normal weight (3–5). These people are usually in good health. In general, intentional weight losses have been found to be protective or unrelated to risk (6, 7). Furthermore, body weight may fluctuate for a variety of other reasons; for instance, various diseases also can cause unintentional weight fluctuation which is associated with smoking, aging and severity of the disease (8).The association between weight fluctuation and health outcomes, especially with mortality and cardiovascular disease (CVD), have been extensively studied since the 1990s (5–29). Several large studies, such as the Honolulu Heart Program, Framingham Heart Study and Treating to New Targets trial, suggested that weight fluctuation is associated with an increased risk of all-cause and CVD mortality (5, 8, 9). Epidemical studies have shown that body weight fluctuations may be associated with metabolic disorders, resulting in negative health consequences (30, 31). However, other cohort studies have failed to confirm these findings (6, 7, 10), or even get opposite results in mild weight fluctuation (6). The association between body-mass index (BMI) and risk of premature death in older people is more complicated (10). A systematic review of cohort studies in populations aged more than 65 years found that either BMI was not a risk factor or low rather than high BMI values increased the risk of all-cause mortality (32). Older people may be more susceptible to unintentional weight fluctuation due to underlying disease. To clarify the association between weight fluctuation and risk for death and CVD, we conducted a systematic review and meta-analysis of the available evidence from published cohort studies.

## Methods

A meta-analysis and meta-regression analyses were carried out for clarifying the association between body-weight fluctuation and risk for mortality and CVD, according to Meta-analysis Of Observational Studies in Epidemiology (MOOSE) Reporting Guidelines (33).

### Search Strategy

We conducted a literature search in PubMed, EMBASE and the Cochrane library from inception to October 15, 2018. Then, manual searching was conducted according to the references of relevant acquired articles. The search was later updated to May 20, 2019. No newly identified study was included in the analyses. Details of the search strategy and data extraction are shown in Appendix S1.

### Study Selection

We included cohort studies conducted in aged 18 years or older and documented exposure to body-weight fluctuation. Weight fluctuation was defined as weight gain or loss in a specific period, and change in the opposite direction (loss or gain) in the next period. It was measured by a continuous variable (i.e., coefficient of variation or root mean square error) and/or a categorical variable (i.e., weight cycle). The stable weight category or lowest category for continuous variables of weight fluctuation was used as the reference. The primary outcomes were risks for mortality from all causes, CVD and cancer, and the secondary outcome was the risk for morbidity of CVD and hypertension. Publications that provided adjusted or crude relative risk (RR) estimates such as risk ratios, incidence rate ratios, hazard ratios or odds ratios with 95% confidence intervals (CI) for weight fluctuation were eligible. Only articles published in English were considered. Multiple publications from the same cohort study were reviewed, and only the papers with the longest follow-up for identical outcomes were included.

We contacted the authors if the data of interest were not directly provided in the publications and got no response.

### Data Extraction

From each eligible study, we extracted data concerning the name of the first author, year of publication, sample size, study design, follow-up duration, definition for weight fluctuation, RRs (95% CIs) for risk of events, and covariates in fully adjusted model (Table A1).

### Statistical Methods

The associations of body-weight fluctuation with health outcomes were assessed, and any results stratified by sex were treated as two separate reports (34). The weight fluctuation was measured using different ways, either as categorical or continuous variables, in publications. For categorical variables, weight fluctuation was defined as weight variation > 4% (11), 4.5% (12), ≥5% (13–15), 10% (16) or others (6, 17–21) of baseline body weight. For continuous variables, root mean square error (RMSE), coefficient of variation (CV), intrapersonal standard deviation of weight (ISD) or average successive variability of weight (ASVW) were used as indexes for weight fluctuation. Body-weight fluctuation, therefore, was measured in two different ways, i.e., weight cycles as categorical variables and deviation degree of weight as continuous variables. For analysis of categorical variables, the risks of outcomes were evaluated by comparing individuals with weight cycles to individuals with stable weight. For analysis of continuous variables, RRs were differently reported by each study (such as per unit or per 1- standard deviation (SD) change, or comparing tertile, quartiles, quintiles or others). For the variables presented as tertile (8, 22), quartiles (23–26) or quintiles (5, 9), the risks of outcomes were evaluated by comparing the top category with bottom category. However, for the RRs of per unit increase of continues variables, such as RR for 1 kg of RMSE or ASVW, the data cannot be transformed to categorical variables and these studies were excluded from our analysis. Therefore, all these RRs for outcomes were treated as categorical variables. We performed a meta-analysis using the random-effects model to calculate RRs for the measurements provided in publications. In the analyses of CVD, risk estimates of two or more kinds of CVD were also treated as separate reports. When publications reported RRs for two different measurements (continues and category variables) simultaneously, we chose the RR calculated from continuous variable since weight cycle may lack some important information and thus has limited power to detect associations (35).

Study quality was assessed using a modified Newcastle-Ottawa scale (NOS) (details are shown in Appendix S2) (36). This scale awards nine scores to each study: four for selection of participants and measurement of exposure, two for comparability of cohorts on the basis of the design or analysis, and three for assessment of outcomes and adequacy of follow-up. A study was considered high quality if it had a score of 6.5 or more. Publication bias was assessed using both Egger's and Begg's tests (37, 38). We also followed the Duval and Tweedie trim and fill procedure as a method of adjustment for suspected publication bias (39). Sensitivity analyses were conducted by omitting 1 report at a time from the analyses and assessing the effect on the overall findings. Subgroup analysis were conducted based on age, measurement of weight fluctuation, method for weight ascertainment, intentional or unintentional weight fluctuation, BMI and adjustment confounding factors. Intentional or unintentional weight change was determined by asking participants if they have lost or gained weight on purpose in questionnaires (6, 7). Heterogeneity was assessed using the *I*^2^ statistic, where *I*^2^ > 50% indicated substantial heterogeneity (40). Meta-regression analyses were conducted to investigate sources of heterogeneity. In the meta-regression, variables in univariable analyses with *P*-values < 0.1 were considered statistically significant and included in multivariable models, and an overall *P*-value < 0.05 was considered statistically significant in multivariable models (41, 42). If there were fewer than 10 studies that reported the explanatory variable(s) of interest, meta-regression analysis could not be performed due to insufficient data.

The statistical analyses were conducted using Stata statistical software version 12.0. A 2-sided *P* < 0.05 was considered to indicate statistical significance.

## Results

### Study Selection

**Figure 1:**
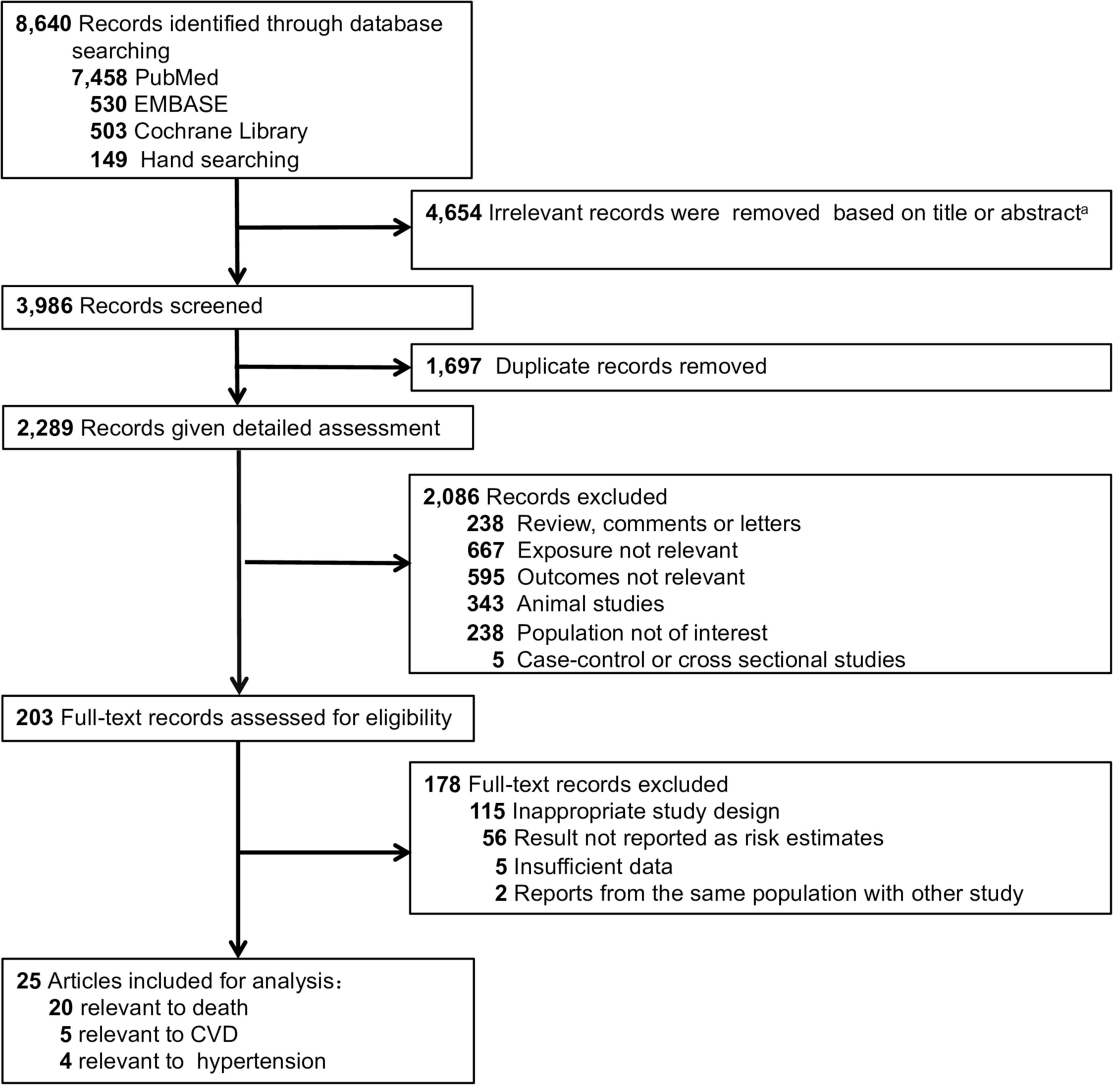
Flow diagram of study selection process. A Exact reasons for exclusions were not documented.

After ineligible studies were excluded from the 8,640 studies identified by the initial search, 23 cohort studies [25 publications (5–29)] were included in our meta-analysis (Figure 1). Among the 25 publications (23 studies), 20 studies provided statistical results relevant to the meta-analyses on mortality, 5 studies on CVD and 4 studies on hypertension.

### Study Characteristics

In 23 studies with 441,199 participants, there were 43,758 deaths due to all causes, 11,721 deaths from CVD, 10,172 deaths from cancer, 15,125 cases of CVD and 18,276 cases of hypertension. Participants were aged 18–79 years, with more than half being middle-aged or older (≥50 years), and participants had an average BMI ≥ 25 in 14 studies (284, 631 participants, 64.5%) (5–8, 11, 15, 16, 18, 19, 23, 24, 26–28) and a BMI ≥ 30 in 3 studies (78,247 participants, 17.7%) (15, 26, 28). Four studies (6, 7, 21, 22, 29) were conducted with participants with intentional weight fluctuation and two (10, 27) with unintentional weight fluctuation; the cause could not be discriminated based on available information in the remaining studies. Four studies (7, 8, 19, 27) reported results stratified by sex and one (11) study reported results as weight cycling end at weight loss and weight gain, respectively. One study (12) reported RR for weight cycle with low, average and high BMI and one study (28) divided participants into groups of diabetes and non-diabetes. Therefore, 15 reports were generated from 7 studies and added to the other 13 studies, resulting in 28 reports in 20 studies included to analyze the association of weight fluctuation and the risk of all-cause mortality (5–16, 18–20, 23, 24, 26–28). Similar situation also showed in analysis of mortality from CVD and cancer, CVD and hypertension. The durations of the cohort studies ranged from 2 to 32 years, with a median duration of 8 years. Twenty of 25 publications had a high quality, as the results of the study quality assessment (score 0–9) yielded a score of 6.5 or above (Table S1).

### Mortality From All Causes, CVD, and Cancer

**Figure 2:**
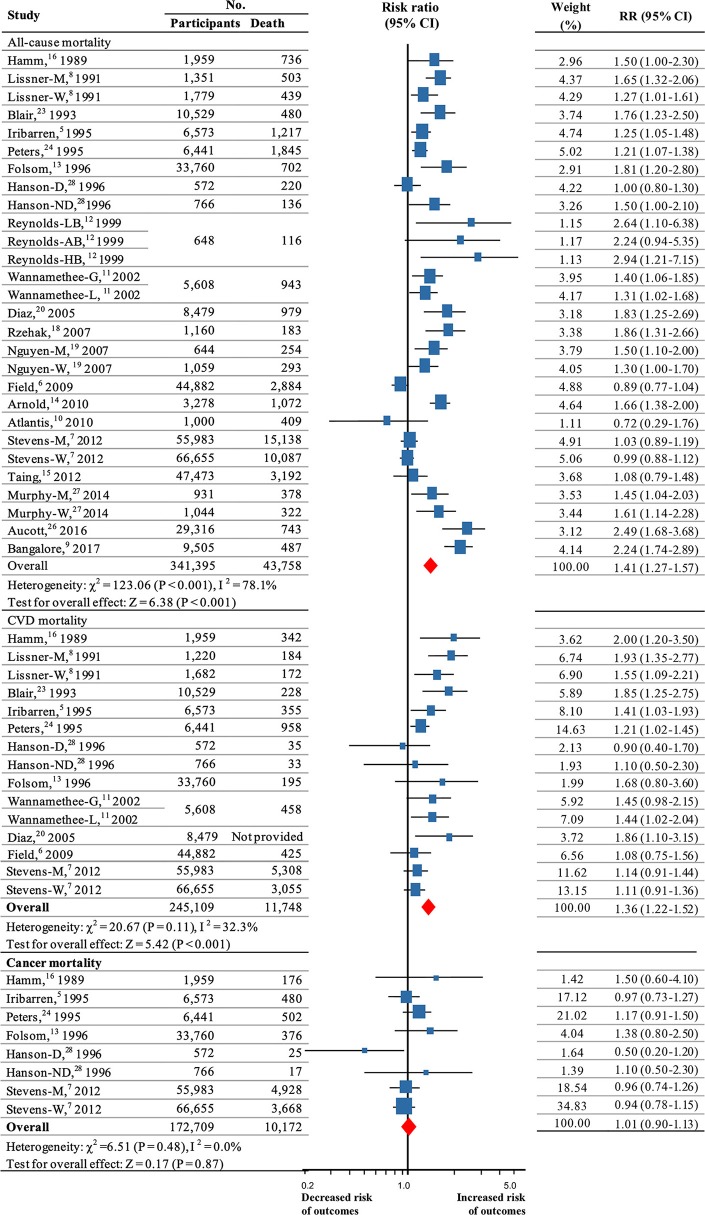
Summary RRs for the association between weight fluctuation and risk all-cause mortality, CVD mortality and cancer mortality. RR and 95% CIs were calculated using the random-effects model used to pool data. Error bars indicate 95% CIs. Risk ratio data are rounded to 2 decimal places; error bars reflect unrounded values. RR, relative risks; CI, confidence interval; CVD, cardiovascular disease. M, men; W, women; G, weight cycle ending with gain; L, weight cycle ending with loss; D, weight fluctuations in diabetes; ND, weight fluctuations in non-diabetes; LB, weight cycle in low BMI population; AB, weight cycle in average BMI population; HB, weight cycle in high BMI population.

Twenty studies were included to analyze the association of weight fluctuation and the risk of all-cause mortality (5–16, 18–20, 23, 24, 26–28).These studies included 43,758 deaths among 341,395 participants. The summary RR for all-cause mortality was 1.41 (95% CI 1.27–1.57; *P* < 0.001; Figure 2) by using a random-effects model (*I*^2^ = 78.1%; *P* < 0.001 for heterogeneity; Table S2).

Eleven (5–8, 11, 13, 16, 20, 23, 24, 28) high-quality studies (245,109 participants, more than 11,748 deaths) were used to evaluate CVD mortality. The results revealed that weight fluctuation was associated with an increased risk for CVD mortality (RR, 1.36, 95% CI 1.22–1.52; *P* < 0.001; Figure 2; *I*^2^ = 32.3%; *P* = 0.11 for heterogeneity; Table S2).

Six high-quality studies (5, 7, 13, 16, 24, 28) (172,709 participants; 10,172 events), were used to evaluate cancer mortality. The analysis showed that weight fluctuation had no influence on cancer mortality (RR, 1.01, 95% CI 0.90–1.13; *P* = 0.87; Figure 2), with no significant heterogeneity among studies (*I*^2^ = 0.0%; *P* = 0.48 for heterogeneity; Table S2).

The results of the sensitivity analyses were not altered after excluding any reports (Figures S1–S3). Furthermore, there were only 3 reports each of intentional and unintentional weight fluctuation in a total of 20 studies with 28 reports for all-cause mortality. Although subgroup analyses showed that unintentional weight fluctuation was associated with increased risk for all-cause mortality (*P* = 0.016; Table S3) and that intentional weight fluctuation was not associated with all-cause mortality (*P* = 0.49; Table S3) or CVD mortality (*P* = 0.12; Table S3). The small number of participants (total participants from 3 reports were 2,975) from unintentional studies limited the conclusion that unintentional weight fluctuation was associated with increased risk for all-cause mortality. Interestingly, the association between weight cycling and all-cause mortality did not vary by BMI or age (all *P* < 0.05). For CVD mortality, a higher risk was observed in normal (*P* = 0.045; Table S3) and overweight individuals (*P* < 0.001; Table S3), but not in those with obesity (BMI ≥ 30) (*P* = 0.82; Table S3) and the old ages (>60 years) (*P* = 0.082; Table S3). Finally, weight fluctuation was associated with increased all-cause and CVD mortality regardless of the way weight fluctuation was measured (all *P* < 0.05; Table S3).

### CVD

**Figure 3:**
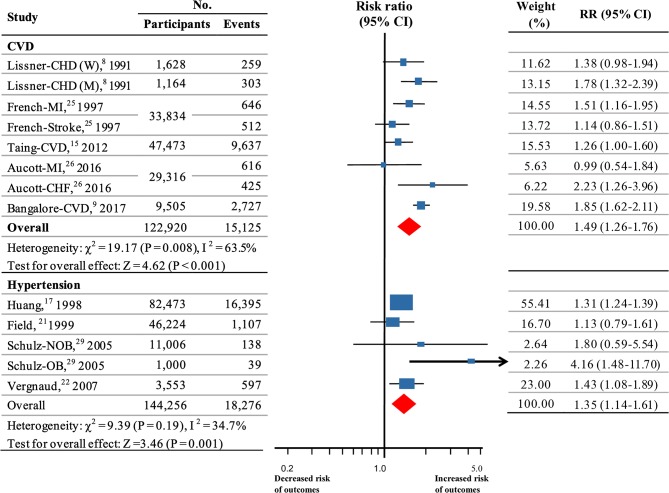
Summary RRs for the association between weight fluctuation and CVD and hypertension. Size of data markers is proportional to the weight of each report. RR and 95% CIs were calculated using the random-effects model used to pool data. Error bars indicate 95% CIs. RR data are rounded to 2 decimal places; error bars reflect unrounded values. RR, relative risks; CI, confidence interval; CVD, cardiovascular disease; M, men; W, women; MI, myocardial infarction; CHF, congestive heart failure; CHD, coronary heart disease; OB, weight fluctuations in obese; NOB, weight fluctuations in non-obese.

Five studies (8, 9, 15, 25, 26), which included a total of 15,125 events among 122,920 participants, were used for the analysis of weight fluctuation and CVD. Three of these studies were of high quality. Pooled estimates showed that weight fluctuation was associated with an increased risk of CVD (RR, 1.49; 95% CI 1.26–1.76; *P* < 0.001; Figure 3) with heterogeneity among studies (*I*^2^ = 63.5%; *P* = 0.008 for heterogeneity; Table S2).

The results of the sensitivity analyses were not altered after excluding any report (Figure S4). However, subgroup analyses for weight fluctuation and risk for CVD showed that separation of methods for weight ascertainment significantly decreased the heterogeneity (*P* = 0.34 for heterogeneity in subgroup of self-reported; *P* = 0.16 for heterogeneity in subgroup of measuring at visit; Table S4), suggesting that mixing different methods of weight ascertainment might be an important source of heterogeneity among the studies of CVD. However, no association was found between weight variation and CVD in obese participants (BMI ≥ 30) (*P* = 0.10; Table S4) as showed for CVD mortality.

### Hypertension

Four studies (17, 21, 22, 29) (144,256 participants, 18,276 events) assessed the association between weight fluctuation and hypertension. All of these participants were in fair health. Our analysis indicated that weight fluctuation was associated with a higher risk for hypertension (RR, 1.35, 95% CI 1.14–1.61; *P* = 0.001; Figure 3). No significant heterogeneity was detected (*I*^2^ = 34.7%; *P* = 0.19 for heterogeneity; Table S2).

In the sensitivity analyses, the results did not change after omitting each of the reports (Figure S5). Although subgroup analyses showed that intentional weight fluctuation was not associated with hypertension (*P* = 0.14; Table S4), however, a limited sample size with 58,230 participants and 1,284 patients restricted the conclusion from the studies.

### Meta-Regression

Substantial heterogeneity (*I*^2^ > 50%) was presented in the analyses of all-cause mortality and cardiovascular morbidity but not in those of CVD and cancer mortality (Table S2); therefore, it was important to investigate the sources of heterogeneity (details in Table S5). In the univariable models various factors were analyzed, study location (*P* = 0.057), duration (*P* = 0.020), study quality (*P* = 0.015), method for weight ascertainment (*P* = 0.005), and adjustment for physical activity (*P* = 0.033) and energy intake (*P* = 0.003) were statistically significant and were therefore eligible for inclusion in the multivariable models for detecting the sources of heterogeneity (all *P* < 0.1).The results from multivariable regression analysis suggested that these factors were the major sources of heterogeneity of the studies for analysis of all-cause mortality (the overall *P* < 0.001 in multivariable models, Table S5). The analysis was not performed among studies for CVD morbidity due to an insufficient number of studies.

### Publication Bias

No evidence of publication bias was observed (all *P* > 0.05) except for studies on all-cause mortality according to the Egger‘s (*P* = 0.001) and Begg's tests (*P* = 0.014) (Table S2). After incorporating the hypothetical studies using trim and fill methods, the risk estimates were attenuated in risk of all-cause mortality (RR, 1.18, 95% CI 1.05–1.32; *P* < 0.001), which suggested the existence of potentially negative studies. Nevertheless, these biases did not change the general conclusion.

## Discussion

In this meta-analysis of 25 studies involving more than 400,000 participants, body-weight fluctuation was associated with a significant increase in risk of all-cause mortality, CVD mortality, and CVD.

A meta-analysis with only 3 studies by Cheng et al. (43) also reported that weight fluctuation in participants aged 60 years or older was associated with a higher risk of mortality, although no detailed subgroup analysis was carried out. Two newly published studies, which didn't include in our analysis for reporting RRs for per unit increase, also concluded that body-weight fluctuation was associated with increased mortality (44, 45). Although from the perspective of the number of studies, 21 of the 28 reports showed that body weight fluctuation was associated with increased risk of all-cause and CVD mortality (8, 9, 11–14, 16, 18–20, 23, 24, 26, 27), the findings from the remaining 7 reports (6, 7, 10, 12, 15, 28), which were equal to more than 60% of both total participants and events in the present study, showed no effect. These observation clearly demonstrated the inconsistency of weight fluctuation and health outcomes. The discrepancy in results may be due to factors such as no differentiation between intentional and unintentional weight fluctuation, participants' age and baseline BMI, and a variety of methods for measuring weight fluctuation. Much of the confusion about the effects of weight fluctuation in studies can be traced to the lack of a standardized definition or measurement for weight fluctuation (1). Subgroup analysis was therefore carried out and showed that regardless of the method of weight fluctuation measurement, its association with all-cause and CVD mortality does exist. There are a variety of reasons for variation in body weight, including subsequent weight regain after intentional weight loss and unintentional weight fluctuation caused by severe diseases (5, 8). Limited studies demonstrated that intentional weight fluctuation was not associated with an increased risk of all-cause and CVD mortality or hypertension in our study. Previous meta-analyses have demonstrated that intentional weight loss is associated with decreased and unintentional weight loss is associated with increased risk of mortality (46–48). It may be caused by the fact that individuals with obesity or overweight are more likely to put on intentional weight-loss regimens, while unintentional weight fluctuation often associated with severity of pre-existing diseases (5, 8), such as edema. However, most studies cannot identify the causes for weight loss. In addition, weight loss is more complicated to study in elderly persons because it is common for older adults to lose muscle mass and their relatively high prevalence of poor health conditions, such as CVD, chronic kidney disease and diabetes (6, 8, 49, 50). The results from our study found that an increased risk for all-cause mortality was observed at all ages and that a higher risk of CVD mortality was observed only in individuals <60 years of age. The results suggest that aging is as important as weight fluctuation for cardiovascular mortality, which is agreeable with previous observation that aging is one of important risk factors for CVD (51). BMI is associated with all-cause mortality and obesity is another of important risk factors for CVD (52–54). Our analysis found that the association with all-cause and CVD mortality occurs in all BMI categories except with CVD mortality in individuals with a BMI≥30. This finding may suggest that obesity had an effect on CVD mortality similar to weight fluctuation.

Fluctuation in body weight was also associated with an increased risk for morbidity of hypertension and CVD and for mortality of CVD, which may account for the increased risk of all-cause mortality. The associations reported here may be interpreted in a number of different ways. One possibility is that weight fluctuation was associated with metabolic disturbance, such as insulin resistance (55), elevations in triglycerides (31) and abdominal fat accumulation (30, 31), and increased risk for diabetes (9, 56–58), all of which may contribute to cardiometabolic disease. Another alternative possibility is that subjects with risk factors for CVD are more likely to experience weight fluctuation (5). In this case, the fluctuation in weight could be the consequence, and not the cause, of the health end points.

To date, no cohort studies have directly compared the clinical outcomes of body-weight fluctuation and maintenance of overweight/obesity or of long-term and stable weight loss. Hence, we cannot conclude whether obese individuals should be deterred from efforts to control their body weight by concerns about the hazards of weight cycling. Future study is needed for the causal links between weight fluctuation and adverse health outcomes.

## Limitations

This analysis has several limitations. First, findings from this review are based on observational data, and no causal links may be concluded. Secondly, different definitions and measurements for weight fluctuation of included studies may be potential confounding variables, although we have conducted detailed subgroup, sensitivity and meta-regression analyses to confirm robustness of our results. And most studies cannot identify intentional or unintentional weight fluctuation, which may have different effect on association between weight fluctuation and health outcomes. Third, the present study did not directly compare clinical outcomes of body-weight fluctuation and maintenance of overweight/obesity or of long-term and stable weight loss. Finally, our results addressed only findings related to changes in weight and no other anthropometric measurements (e.g., waist circumference and waist-to-hip ratio). Loss of lean body mass may be a powerful predictor of increased mortality risk in older persons (20), but such measures are well beyond the scope of our present analyses.

## Conclusion

In conclusion, the present systematic review and meta-analysis revealed that body-weight fluctuation was associated with higher mortality of all causes and CVD and morbidity of CVD and hypertension. Future study is needed for the causal links between weight fluctuation and adverse health outcomes.

## Author Contributions

XY and HZ had full access to all of the data in the study and take responsibility for the integrity of the data and the accuracy of the data analysis. XY designed the study. HZ, ZZ, WLi, WLiu, and QZ selected relevant studies and analysis data. Studies were selected and appraised by two trained clinician reviewers (HZ and ZZ). Data were extracted from included studies using a specially developed data extraction form by two independent reviewers (WLi and QZ). Any disagreement was resolved by discussion with a third review author to reach a consensus (YY). Missing data was obtained from the authors wherever possible. Five authors were contacted for data reanalysis and additional information and none of them replied us and provided additional data. PY, LL, and YY reviewed and provided suggestions. All authors reviewed the manuscript and approved the final manuscript.

### Conflict of Interest

The authors declare that the research was conducted in the absence of any commercial or financial relationships that could be construed as a potential conflict of interest.

## References

[1] National Task Force on the Prevention and Treatment of Obesity Weight cycling. JAMA. (1994) 272:1196–202. 10.1001/jama.272.15.1196 7741844

[2] Toray T Cooley E. Weight fluctuation, bulimic symptoms, and self-efficacy for control of eating. J Psychol Interdisc Appl. (1997) 131:383–92. 10.1080/00223989709603524 9190055

[3] Simkin-Silverman LR Wing RR Plantinga P Matthews KA Kuller LH. Lifetime weight cycling and psychological health in normal-weight and overweight women. Int J Eat Disord. (1998) 24:175–83. 9697016 10.1002/(sici)1098-108x(199809)24:2<175::aid-eat7>3.0.co;2-b

[4] Field AE Wing RR Manson JE Spiegelman DL Willett WC. Relationship of a large weight loss to long-term weight change among young and middle-aged US women. Int J Obes. (2001) 25:1113–21. 10.1038/sj.ijo.0801643 11477495

[5] Iribarren C Sharp DS Burchfiel CM Petrovitch H. Association of weight loss and weight fluctuation with mortality among Japanese American men. New Engl J Med. (1995) 333:686–92. 10.1056/NEJM199509143331102 7637745

[6] Field AE Malspeis S Willett WC Weight cycling and mortality among middle-aged or older women. Arch Int Med. (2009) 169:881 10.1001/archinternmed.2009.67 19433700 PMC2764352

[7] Stevens VL Jacobs EJ Sun J Patel AV McCullough ML Teras LR. Weight cycling and mortality in a large prospective US study. Am J Epidemiol. (2012) 175:785. 10.1093/aje/kwr378 22287640

[8] Lissner L Odell PM D'Agostino RB Stokes J Kreger BE Belanger AJ. Variability of body weight and health outcomes in the Framingham population. New Engl J Med. (1991) 324:1839–44. 10.1056/NEJM199106273242602 2041550

[9] Bangalore S Fayyad R Laskey R DeMicco DA Messerli FH Waters DD. Body-weight fluctuations and outcomes in coronary disease. N Engl J Med. (2017) 376:1332–40. 10.1056/NEJMoa1606148 28379800

[10] Atlantis E Browning C Kendig H. Body mass index and unintentional weight change associated with all-cause mortality in older Australians: the Melbourne Longitudinal Studies on Healthy Ageing (MELSHA). Age Ageing. (2010) 39:643–6. 10.1093/ageing/afq073 20584733

[11] Wannamethee SG Shaper AG Walker M. Weight change, weight fluctuation, and mortality. Arch Int Med. (2002) 162:2575. 10.1001/archinte.162.22.2575 12456229

[12] Reynolds MW Fredman L Langenberg P Magaziner. Weight, weight change, and mortality in a random sample of older community-dwelling women. J Am Geriatr Soc. (1999) 47:1409–14. 10.1111/j.1532-5415.1999.tb01558.x 10591233

[13] Folsom AR French SA Zheng W Baxter JE Jeffery RW. Weight variability and mortality: the Iowa Women's Health Study. Int J Obesity. (1996) 20:704. 8856391

[14] Arnold AM Newman AB Cushman M Ding J Kritchevsky S. Body weight dynamics and their association with physical function and mortality in older adults: the cardiovascular health study. J Gerontol. (2010) 65:63. 10.1093/gerona/glp050 19386574 PMC2796878

[15] Taing KY Ardern CI Kuk JL. Effect of the timing of weight cycling during adulthood on mortality risk in overweight and obese postmenopausal women. Obesity. (2012) 20:407–13. 10.1038/oby.2011.207 21760629

[16] Hamm P Shekelle RB Stamler J. Large fluctuations in body weight during young adulthood and twenty-five-year risk of coronary death in men. Am J Epidemiol. (1989) 129:312–8. 10.1093/oxfordjournals.aje.a115135 2912043

[17] Huang Z Willett WC Manson JE Rosner B Stampfer MJ Speizer FE. Body weight, weight change, and risk for hypertension in women. Ann Intern Med. (1998) 128:81–8. 10.7326/0003-4819-128-2-199801150-00001 9441586

[18] Rzehak P Meisinger C Woelke G Brasche S Strube G Heinrich J. Weight change, weight cycling and mortality in the ERFORT Male Cohort Study. Eur J Epidemiol. (2007) 22:665–73. 10.1007/s10654-007-9167-5 17676383

[19] Nguyen ND Center JR Eisman JA Nguyen TV. Bone loss, weight loss, and weight fluctuation predict mortality risk in elderly men and women. J Bone Miner Res. (2007) 22:1147–54. 10.1359/jbmr.070412 17635040

[20] Diaz VA Mainous AG Everett CJ. The association between weight fluctuation and mortality: results from a population-based cohort study. J Commun Health. (2005) 30:153–65. 10.1007/s10900-004-1955-1 15847242

[21] Field AE Byers T Hunter DJ Laird NM Manson JE Williamson DF. Weight cycling, weight gain, and risk of hypertension in women. Am J Epidemiol. (1999) 150:573–9. 10.1093/oxfordjournals.aje.a010055 10489996

[22] Vergnaud AC Bertrais S Oppert JM Maillard-Teyssier L Galan P Hercberg S. Weight fluctuations and risk for metabolic syndrome in an adult cohort. Int J Obesity. (2007) 32:315. 10.1038/sj.ijo.0803739 17968381

[23] Blair SN Shaten J Brownell K Collins G Lissner L. Body weight change, all-cause mortality, and cause-specific mortality in the Multiple Risk Factor Intervention Trial. Ann Intern Med. (1993) 119:749–57. 10.7326/0003-4819-119-7_Part_2-199310011-00024 8363210

[24] Peters ET Seidell JC Menotti A Arayanis C Dontas A Fidanza F. Changes in body weight in relation to mortality in 6441 European middle-aged men: the Seven Countries Study. Int J Obesity. (1995) 19:862–8. 8963353

[25] French SA Folsom AR Jeffery RW Zheng W Mink PJ Baxter JE. Weight variability and incident disease in older women: the Iowa Women's Health Study. Int J Obesity. (1997) 21:217. 10.1038/sj.ijo.0800390 9080261

[26] Aucott LS Philip S Avenell A Afolabi E Sattar N Wild S. Patterns of weight change after the diagnosis of type 2 diabetes in Scotland and their relationship with glycaemic control, mortality and cardiovascular outcomes: a retrospective cohort study. BMJ Open. (2016) 6:e10836. 10.1136/bmjopen-2015-010836 27466237 PMC4964186

[27] Murphy RA Patel KV Kritchevsky SB Houston DK Newman AB Koster A. Weight change, body composition, and risk of mobility disability and mortality in older adults: a population-based cohort study. J Am Geriatr Soc. (2014) 62:1476–83. 10.1111/jgs.12954 25039391 PMC4134405

[28] Hanson RL Jacobsson LT Mccance DR Narayan KM Pettitt DJ Bennett PH. Weight fluctuation, mortality and vascular disease in Pima Indians. Int J Obesity. (1996) 20:463. 8696426

[29] Schulz M Liese AD Boeing H Cunningham JE Moore CG Kroke A. Associations of short-term weight changes and weight cycling with incidence of essential hypertension in the EPIC-Potsdam Study. J Hum Hypertens. (2005) 19:61–7. 10.1038/sj.jhh.1001776 15343355

[30] Rodin J Radke-Sharpe N Rebuffé-Scrive M Greenwood MR. Weight cycling and fat distribution. Int J Obes. (1990) 14:303–10. 2361807

[31] Cereda E Malavazos AE Caccialanza R Rondanelli M Fatati G Barichella M. Weight cycling is associated with body weight excess and abdominal fat accumulation: a cross-sectional study. Clin Nutr. (2011) 30:718–23. 10.1016/j.clnu.2011.06.009 21764186

[32] Heiat A Vaccarino V Krumholz HM. An evidence-based assessment of federal guidelines for overweight and obesity as they apply to elderly persons. Arch Intern Med. (2001) 161:1194–203. 10.1001/archinte.161.9.1194 11343442

[33] Stroup DF Berlin JA Morton SC Olkin I Williamson GD Rennie D. Meta-analysis of observational studies in epidemiology: a proposal for reporting. Meta-analysis Of Observational Studies in Epidemiology (MOOSE) group. JAMA. (2000) 283:2008–12. 10.1001/jama.283.15.2008 10789670

[34] Rong Y Chen L Zhu T Song Y Yu M Shan Z. Egg consumption and risk of coronary heart disease and stroke: dose-response meta-analysis of prospective cohort studies. BMJ. (2013) 346:1–13. 10.1136/bmj.e8539 23295181 PMC3538567

[35] French SA Jeffery RW Folsom AR Williamson DF Byers T Weight variability in a population-based sample of older women: reliability and inter correlation of measures. Int J Obesity. (1995) 19:22. 7719387

[36] Wells GA Shea B O'Connell D Peterson J Welch V Losos M The Newcastle-Ottawa Scale (NOS) for Assessing the Quality of Nonrandomized Studies in Meta-Analyses. (2011). Available online at: www.ohri.ca/programs/clinical_epidemiology/oxford.asp

[37] Egger M Davey SG Schneider M Minder C. Bias in meta-analysis detected by a simple, graphical test. BMJ. (1997) 315:629–34. 10.1136/bmj.315.7109.629 9310563 PMC2127453

[38] Begg CB Mazumdar M. Operating characteristics of a rank correlation test for publication bias. Biometrics. (1994) 50:1088–101. 10.2307/2533446 7786990

[39] Duval S Tweedie R. Trim and fill: A simple funnel-plot-based method of testing and adjusting for publication bias in meta-analysis. Biometrics. (2000) 56:455–63. 10.1111/j.0006-341X.2000.00455.x 10877304

[40] Higgins JP Thompson SG Deeks JJ Altman DG. Measuring inconsistency in meta-analyses. BMJ. (2003) 327:557–60. 10.1136/bmj.327.7414.557 12958120 PMC192859

[41] Samprit Chatterjee ASH Regression Analysis by Example. 4th edn New York, NY: Wiley-Interscience (2006). 10.1002/0470055464

[42] Thompson SG Higgins JPT. How should meta-regression analyses be undertaken and interpreted? Stat Med. (2002) 21:1559–73. 10.1002/sim.1187 12111920

[43] Cheng FW Gao X Jensen GL. Weight change and all-cause mortality in older adults: a meta-analysis. J Nutr Gerontol Geriatr. (2015) 34:343–68. 10.1080/21551197.2015.1090362 26571354

[44] Oh TJ Moon JH Choi SH Lim S Park KS Cho NH. Body-weight fluctuation and incident diabetes mellitus, cardiovascular disease, and mortality: a 16-year prospective cohort study. J Clin Endocrinol Metab. (2019) 104:639–46. 10.1210/jc.2018-01239 30500906

[45] Cologne J Takahashi I French B Nanri A Misumi M Sadakane A. Association of weight fluctuation with mortality in Japanese adults. JAMA Netw Open. (2019) 2:e190731. 10.1001/jamanetworkopen.2019.0731 30874785 PMC6484619

[46] Kritchevsky SB Beavers KM Miller ME Shea MK Houston DK Kitzman DW. Intentional weight loss and all-cause mortality: a meta-analysis of randomized clinical trials. PLoS ONE. (2015) 10:e0121993. 10.1371/journal.pone.0121993 25794148 PMC4368053

[47] Harrington M Gibson S Cottrell RC. A review and meta-analysis of the effect of weight loss on all-cause mortality risk. Nutr Res Rev. (2009) 22:93–108. 10.1017/S0954422409990035 19555520

[48] De Stefani FC Pietraroia PS Fernandes-Silva MM Faria-Neto J Baena CP. Observational evidence for unintentional weight loss in all-cause mortality and major cardiovascular events: a systematic review and meta-analysis. Sci Rep. (2018) 8:1–11. 10.1038/s41598-018-33563-z 30337578 PMC6194006

[49] Sundquist J Winkleby MA Pudaric S. Cardiovascular Disease Risk Factors among Older Black, Mexican-American, and white women and men: an analysis of NHANES III, 1988–1994. J Am Geriatr Soc. (2001) 49:109–16. 10.1046/j.1532-5415.2001.49030.x 11207863

[50] Svetkey LP Clark JM Funk K Corsino L Batch BC Hollis JF. Greater weight loss with increasing age in the weight loss maintenance trial. Obesity. (2014) 22:39–44. 10.1002/oby.20506 23640912 PMC3849225

[51] Chia CW Egan JM Ferrucci L. Age-related changes in glucose metabolism, hyperglycemia, and cardiovascular risk. Circ Res. (2018) 123:886–904. 10.1161/CIRCRESAHA.118.312806 30355075 PMC6205735

[52] Flegal KM Kit BK Orpana H. Association of all-cause mortality with overweight and obesity using standard body mass index categories: a systematic review and meta-analysis. JAMA. (2013) 309:71–82. 10.1001/jama.2012.113905 23280227 PMC4855514

[53] Bhaskaran K dos-Santos-Silva I Leon DA Douglas IJ Smeeth L. Association of BMI with overall and cause-specific mortality: a population-based cohort study of 3.6 million adults in the UK. Lancet Diabetes Endocrinol. (2018) 6:944–53. 10.1016/S2213-8587(18)30288-2 30389323 PMC6249991

[54] Poirier P Giles TD Bray GA Hong Y Stern JS Pi-Sunyer FX. Obesity and cardiovascular disease: pathophysiology, evaluation, and effect of weight loss : an update of the 1997 American heart association scientific statement on obesity and heart disease from the obesity committee of the council on nutrition, physical. Circulation. (2006) 113:898–918. 10.1161/CIRCULATIONAHA.106.171016 16380542

[55] Anastasiou CA Yannakoulia M Pirogianni V Rapti G Sidossis LS Kavouras SA. Fitness and weight cycling in relation to body fat and insulin sensitivity in normal-weight young women. J Am Dietetic Assoc. (2010) 110:280–4. 10.1016/j.jada.2009.10.040 20102857

[56] Rhee E Cho JH Kwon H Park SE Park CY Oh KW Increased risk of diabetes development in individuals with weight cycling over 4 years: The Kangbuk Samsung Health study. Diabetes Res Clin Pr. (2018) 139:230–8. 10.1016/j.diabres.2018.03.018 29574105

[57] Holbrook TL Barrett-Connor E Wingard DL. The association of lifetime weight and weight control patterns with diabetes among men and women in an adult community. Int J Obesity. (1989) 13:723–9. 2583926

[58] Kataja-Tuomola M Sundell J Männistö S Virtanen MJ Kontto J Albanes D. Short-term weight change and fluctuation as risk factors for type 2 diabetes in Finnish male smokers. Eur J Epidemiol. (2010) 25:333–9. 10.1007/s10654-010-9444-6 20352298

