# Supplementary material for: Body-Weight Fluctuation Was Associated With Increased Risk for Cardiovascular Disease, All-Cause and Cardiovascular Mortality: A Systematic Review and Meta-Analysis
Source: Front Endocrinol (Lausanne). 2019 Nov 8;10:728. doi: 10.3389/fendo.2019.00728 (PMC6856014; doi:10.3389/fendo.2019.00728)
Supplement: Supplementary file 1 [file Presentation_1.pdf]

Supplementary Online Content

Huajie Zou, Ping Yin, et al. Body-weight Fluctuation was Associated with Increased risk for Cardiovascular Disease, All-cause and Cardiovascular Mortality: A Systematic Review and Meta-analysis

Appendix 1. Search Terms
Appendix 2. MOOSE Checklist for Meta-analyses of Observational Studies
Table S1. Quality assessment of individual studies using Newcastle-Ottawa Scale
Table S2. Assessment for heterogeneity and publication bias
Table S3. Subgroup analyses for association between weight fluctuation and risk of mortality
Table S4. Subgroup analyses for association between weight fluctuation and risk of CVD and hypertension
Table S5. Meta-regression of factors affecting heterogeneity of studies for all-cause mortality
Figure S1. Sensitivity analysis for association of weight fluctuation and risk of all-cause mortality
Figure S2. Sensitivity analysis for association of weight fluctuation and risk of CVD mortality
Figure S3. Sensitivity analysis for association of weight fluctuation and risk of cancer mortality
Figure S4. Sensitivity analysis for association of weight fluctuation and risk of CVD
Figure S5. Sensitivity analysis for association of weight fluctuation and risk of hypertension
Online Reference.

Appendix 1. Search Terms

Appendix 1. 1. Search Terms in PubMed

#1 "fluctuation"[All Fields] OR "variability"[All Fields] OR "regain"[All Fields] OR "maintenance"[All Fields] OR "maintain"[All Fields] OR "cycling"[All Fields] OR "cycle"[All Fields]

#2 "weight"[All Fields] OR "body mass"[All Fields] OR "BMI"[All Fields]

#3 #1 AND #2

#4 "mortality"[MeSH Terms] OR "mortality"[All Fields] OR "death"[All Fields] OR " fatal"[All Fields]

#5 #3 AND #4

#6 "cardiovascular diseases"[MeSH Terms] OR ("cardiovascular"[All Fields] AND "diseases"[All Fields]) OR "cardiovascular diseases"[All Fields] OR ("cardiovascular"[All Fields] AND "disease"[All Fields]) OR "cardiovascular disease"[All Fields]

#7 #3 AND #6

#8 #5 OR #7

#10 limit 8 to (Humans)

#11 limit 10 to English [Language]

Appendix 1. 2. Search Terms in EMBASE

#1 "fluctuation" OR "variability" OR "regain" OR "maintenance" OR "maintain" OR "cycling" OR "cycle"

#2 "weight" OR "body mass" OR "BMI"

#3 #1 AND #2

#4 "mortality" OR "death" OR " fatal"

#5 #3 AND #4

#6 "cardiovascular diseases"

#7 #5 AND #6

#8 #5 OR #7

#9 "cohort study" OR "longitudinal study" OR “prospective study"

#10 #8 AND #9

#11 limit 10 to (Humans)

#12 limit 11 to English [Language]

Appendix 1. 3. Search Terms in Cochrane Library.

#1 "fluctuation" or "variability" or "regain" or "maintenance" or "maintain" or "cycling" or "cycle"

#2 "weight" or "body mass" or "BMI"

#3 #1 and #2

#4 "mortality" or "death" or " fatal"

#5 #3 and #4

#6 "cardiovascular diseases"

#7 #5 and #6

#8 #5 or #7

#9 "cohort study" or "longitudinal study" or “prospective study"

#10 #8 and #9

Appendix 2. MOOSE Checklist for Meta-analyses of Observational Studies

Item No	Recommendation	Reported on Page No
Reporting of background should include		
1	Problem definition	2, 4-5
2	Hypothesis statement	2
3	Description of study outcome(s)	4
4	Type of exposure or intervention used	3
5	Type of study designs used	4
6	Study population	3
Reporting of search strategy should include		
7	Qualifications of searchers (eg, librarians and investigators)	None
8	Search strategy, including time period included in the synthesis and key words	3
9	Effort to include all available studies, including contact with authors	4
10	Databases and registries searched	3
11	Search software used, name and version, including special features used (eg, explosion)	3
12	Use of hand searching (eg, reference lists of obtained articles)	3
13	List of citations located and those excluded, including justification	Figure 1
14	Method of addressing articles published in languages other than English	None
15	Method of handling abstracts and unpublished studies	3
16	Description of any contact with authors	4
Reporting of methods should include		
17	Description of relevance or appropriateness of studies assembled for assessing the hypothesis to be tested	3
18	Rationale for the selection and coding of data (eg, sound clinical principles or convenience)	4
19	Documentation of how data were classified and coded (eg, multiple raters, blinding and interrater reliability)	16
20	Assessment of confounding (eg, comparability of cases and controls in studies where appropriate)	Supplement-7
21	Assessment of study quality, including blinding of quality assessors, stratification or regression on possible predictors of study results	5-6
22	Assessment of heterogeneity	6
23	Description of statistical methods (eg, complete description of fixed or random effects models, justification of whether the chosen models account for predictors of study results, dose-response models, or cumulative meta-analysis) in sufficient detail to be replicated	5-6
24	Provision of appropriate tables and graphics	Table, figures and supplement file
Reporting of results should include		

25	Graphic summarizing individual study estimates and overall estimate	Figure2-3
26	Table giving descriptive information for each study included	Table 1
27	Results of sensitivity testing (eg, subgroup analysis)	Supplement 9-18
28	Indication of statistical uncertainty of findings	None

Item No	Recommendation	Reported on Page No
Reporting of discussion should include		
29	Quantitative assessment of bias (eg, publication bias)	11
30	Justification for exclusion (eg, exclusion of non-English language citations)	Figure1
31	Assessment of quality of included studies	Supplement-7
Reporting of conclusions should include		
32	Consideration of alternative explanations for observed results	14
33	Generalization of the conclusions (ie, appropriate for the data presented and within the domain of the literature review)	15
34	Guidelines for future research	14
35	Disclosure of funding source	16

From: Stroup DF, Berlin JA, Morton SC, et al, for the Meta-analysis Of Observational Studies in Epidemiology (MOOSE) Group. Meta-analysis of Observational Studies in Epidemiology. A Proposal for Reporting. *JAMA*. 2000; 283(15):2008-2012. doi: 10.1001/jama.283.15.2008.

Transcribed from the original paper within the NEUROSURGERY® Editorial Office, Atlanta, GA, United Sates. August 2012.

Table S1. Quality assessment of individual studies using Newcastle-Ottawa Scale

Reference	Selection				Comparability	Outcome			Overall quality
	Representative of cases	Selection of controls	Exposure ascertainment (weight change)	No history of disease	Comparable on confounders	Outcome assessment (by medical record or doctors)	Adequate follow-up time (≥ 5 years)	Follow-up rate (> 80%)	
Hamm, et al[1]	0.5	1.0	1.0	1.0	1.0	0.5	1.0	1.0	7.0
Lissner, et al[2]	1.0	1.0	1.0	1.0	2.0	1.0	1.0	0.0	8.0
Blair, et al[3]	0.5	1.0	1.0	1.0	1.0	1.0	1.0	1.0	6.5
Iribarren, et al[4]	0.5	1.0	1.0	1.0	2.0	0.5	1.0	1.0	8.0
Peters, et al[5]	1.0	1.0	0.0	1.0	2.0	1.0	1.0	0.0	7.0
Folsom, et al[6]	0.5	1.0	1.0	1.0	2.0	1.0	1.0	1.0	8.5
French, et al[7]	0.5	1.0	1.0	1.0	2.0	1.0	1.0	1.0	8.5
Hanson, et al[8]	1.0	1.0	1.0	1.0	2.0	1.0	1.0	1.0	9.0
Reynolds, et al[9]	0.5	1.0	1.0	1.0	2.0	1.0	1.0	1.0	8.5
Wannamethee, et al[10]	0.5	1.0	0.5	1.0	2.0	0.5	1.0	1.0	7.0
Diaz, et al[11]	1.0	1.0	0.5	1.0	1.0	1.0	1.0	0.0	6.5
Nguyen, et al[12]	0.5	1.0	0.5	1.0	2.0	1.0	1.0	0.0	7.0
Rzehak, et al[13]	0.5	1.0	1.0	1.0	0.5	1.0	1.0	0.0	6.0
Huang, et al[14]	0.5	1.0	1.0	1.0	1.0	1.0	1.0	1.0	7.5
Field, et al[15]	0.5	1.0	0.5	1.0	1.0	1.0	1.0	0.0	6.0
Field, et al[16]	0.5	1.0	1.0	1.0	2.0	1.0	1.0	0.0	7.5
Arnold, et al[17]	1.0	1.0	0.5	1.0	0.5	1.0	1.0	1.0	7.0
Atlantis, et al[18]	0.5	1.0	0.5	1.0	1.0	1.0	1.0	0.0	6.0
Taing, et al[19]	0.5	1.0	1.0	1.0	1.0	0.0	1.0	0.0	5.5
Stevens, et al[20]	1.0	1.0	0.5	1.0	2.0	1.0	1.0	0.0	7.5
Murphy, et al[21]	0.5	1.0	1.0	1.0	2.0	1.0	1.0	0.0	7.5
Aucott, et al[22]	0.5	1.0	1.0	0.5	1.0	1.0	1.0	0.0	6.0
Bangalore, et al[23]	0.5	1.0	0.5	1.0	2.0	1.0	0.0	1.0	6.5
Schulz, et al[24]	1.0	1.0	1.0	1.0	1.0	1.0	0.0	1.0	7.0
Vergnaud, et al[25]	0.5	1.0	1.0	1.0	1.0	1.0	1.0	0.0	6.5

Table S2. Assessment for heterogeneity and publication bias

	Tests for Heterogeneity				Tests for Publication Bias		
	RR (95% CI) by random-effect model	RR (95% CI) by fixed-effect model	P value for heterogeneity	I^2 (%)	P value of the Egger's test	P value of the Begg's test	Trim & Fill RR (95% CI)
**Primary outcomes**							
All-cause mortality	1.41 (1.27-1.57)	1.26 (1.20-1.32)	< 0.001	78.1	0.001	0.014	1.18 (1.05-1.32)
CVD mortality	1.36 (1.22-1.52)	1.31 (1.21-1.43)	0.11	32.3	0.068	0.17	1.24 (1.09-1.40)
Cancer mortality	1.01 (0.90-1.13)	1.01 (0.90-1.13)	0.48	0.0	0.68	0.90	1.00 (0.90-1.13)
**Secondary outcomes**							
CVD	1.49 (1.26-1.76)	1.58 (1.45-1.73)	0.008	63.5	0.24	0.90	1.49 (1.26-1.76)
Hypertension	1.35 (1.14-1.61)	1.32 (1.25-1.39)	0.19	34.7	0.34	0.81	1.30 (1.05-1.62)

Abbreviations: RR, relative risk; CI, confidence intervals; CVD, cardiovascular diseases.

Table 3. Subgroup analyses of relative risk of mortality.

		All-cause mortality					CVD mortality				
		n	RR (95% CI)	P_1	I_2 (%)	P_2	n	RR (95% CI)	P_1	I_2 (%)	P_2
All studies		28	1.41 (1.27-1.57)	< 0.001	78.1	< 0.001	15	1.36 (1.22-1.52)	< 0.001	32.3	0.11
Age											
> 60 years		14	1.45 (1.21-1.74)	< 0.001	80.9	< 0.001	3	1.14 (0.98-1.32)	0.082	0.0	0.58
≤ 60 years		14	1.39 (1.21-1.59)	< 0.001	76.4	< 0.001	12	1.44 (1.27-1.63)	< 0.001	24.5	0.20
Duration (years)											
> 8		14	1.25 (1.10-1.43)	0.001	75.6	< 0.001	9	1.38 (1.17-1.64)	< 0.001	46.3	0.06
≤ 8		14	1.61 (1.39-1.85)	< 0.001	67.0	< 0.001	6	1.33 (1.15-1.52)	< 0.001	13.2	0.33
Measurement of weight fluctuation											
Deviations degree		8	1.54 (1.26-1.88)	< 0.001	79.2	< 0.001	8	1.47 (1.24-1.73)	< 0.001	35.7	0.14
Weight cycle		20	1.35 (1.20-1.53)	< 0.001	74.7	< 0.001	7	1.25 (1.09-1.43)	0.002	16.7	0.30
Method for weight ascertainment											
Self-reported		9	1.13 (0.99-1.30)	0.073	64.6	0.004	7	1.25 (1.09-1.43)	0.002	16.7	0.30
Measured at each visit		19	1.55 (1.38-1.74)	< 0.001	66.0	< 0.001	8	1.47 (1.24-1.73)	< 0.001	35.7	0.14
Weight loss											
Intentional		3	0.97 (0.90-1.05)	0.49	1.1	0.36	3	1.12 (0.97-1.28)	0.12	0.0	0.97
Unintentional		3	1.42 (1.07-1.89)	0.016	25.0	0.26	None				
No discrimination		22	1.51 (1.36-1.67)	< 0.001	63.4	< 0.001	12	1.47 (1.30-1.65)	< 0.001	13.1	0.32
BMI											
BMI < 25		6	1.54 (1.14-2.09)	0.005	78.0	< 0.001	3	1.35 (1.01-1.81)	0.045	57.3	0.10
25≤ BMI <30		16	1.32 (1.18-1.48)	< 0.001	70.0	< 0.001	11	1.41 (1.22-1.64)	< 0.001	43.4	0.06
BMI ≥ 30		4	1.49 (1.01-2.20)	0.04	72.9	< 0.001	2	1.06 (0.62-1.81)	0.82	0.0	0.90
Adjustment for one of following confounding factors											
Weight change from baseline	Yes	18	1.38 (1.21-1.57)	< 0.001	82.2	< 0.001	11	1.29 (1.14-1.45)	< 0.001	28.5	0.17
	No	10	1.50 (1.29-1.74)	< 0.001	45.2	0.06	4	1.61 (1.32-1.97)	< 0.001	0.0	0.62
Physical activity	Yes	18	1.30 (1.16-1.46)	< 0.001	74.9	< 0.001	11	1.40 (1.22-1.60)	< 0.001	40.2	0.08
	No	10	1.74 (1.37-2.22)	< 0.001	79.5	< 0.001	4	1.24 (1.03-1.50)	0.02	6.3	0.36

Abbreviations: BMI, body-mass index; CI, confidence interval; CVD, cardiovascular disease.

n, the number of studies (the number of studies is not always equal to the total because of missing information in some publications or subgroups in original studies).

P_1 for significance of association of weight fluctuation and risk of outcomes in each subgroup.

P_2 for heterogeneity within each subgroup.

Table S4. Subgroup analyses of relative risk of CVD and hypertension.

		CVD					Hypertension				
		n	RR (95% CI)	P_1	I_2 (%)	P_2	n	RR (95% CI)	P_1	I_2 (%)	P_2
All studies		8	1.49 (1.26-1.76)	< 0.001	63.5	0.008	5	1.35 (1.14-1.69)	0.001	34.7	0.19
Age											
> 60 years		4	1.44 (1.13-1.83)	0.003	79.1	0.002	None				
≤ 60 years		4	1.56 (1.19-2.04)	0.001	38.1	0.18	5	1.35 (1.14-1.69)	0.001	34.7	0.19
Duration											
> 6 years		3	1.44 (1.17-1.78)	0.001	38.4	0.20	4	1.38 (1.17-1.62)	< 0.001	17.3	0.31
≤ 6 years		5	1.50 (1.17-1.93)	0.001	70.8	0.008	3	1.83 (0.82-4.08)	0.14	65.3	0.06
Measurement of weight fluctuation											
Deviations degree		7	1.54 (1.28-1.84)	< 0.001	59.9	0.20	None				
Weight cycle		1	1.26 (0.99-1.59)	0.05			5	1.35 (1.14-1.69)	0.001	34.7	0.19
Method for weight ascertainment											
Self-reported		3	1.30 (1.11-1.52)	< 0.001	8.3	0.34	4	1.38 (1.03-1.85)	0.03	48.0	0.12
Measured at each visit		5	1.68 (1.40-2.02)	< 0.001	38.9	0.16	1	1.43 (1.08-1.89)	0.01	-	-
Weight loss											
Intentional		None					3	1.83 (0.82- 4.08)	0.14	65.3	0.06
Unintentional		None					None				
No discrimination		8	1.49 (1.26-1.76)	< 0.001	63.5	0.008	2	1.32 (1.24-1.39)	< 0.001	0.0	0.55
BMI											
BMI < 25		0					3	1.32 (1.07-1.64)	0.01	0.0	0.51
25≤ BMI <30		2	1.59 (1.24-2.04)	< 0.001	17.8	0.27	0				
BMI ≥ 30		3	1.38 (0.94-2.02)	0.10	52.6	0.008	1	4.16 (1.48-11.70)	< 0.001	-	-
Adjustment for one of following confounding factors											
Weight change from baseline	Yes	5	1.54 (1.28-1.86)	< 0.001	64.5	0.02	1	1.43 (1.08-1.89)	0.01	-	-
	No	3	1.38 (0.94-2.02)	0.10	52.6	0.12	4	1.38 (1.03-1.85)	0.03	48.0	0.12
Physical activity	Yes	5	1.39 (1.20-1.61)	< 0.001	29.0	0.36	5	1.43 (1.17-1.75)	< 0.001	36.1	0.15
	No	3	1.69 (1.18-2.42)	0.004	53.8	0.16	None				

Abbreviations: BMI, body-mass index; CI, confidence interval; CVD, cardiovascular disease.

n, the number of studies (the number of studies is not always equal to the total because of missing information in some publications or subgroups in original studies).

P_1 for significance of association of weight fluctuation and risk of outcomes in each subgroup.

P_2 for heterogeneity within each subgroup.

Table S5. Meta-regression of factors associated with studies for all-cause mortality.

Variable	Coefficient	P-value	Lower CI	Upper CI	I_2	Overall P-value
Sex (male or female or both)	-	0.183	-	-	26.44%	< 0.001
Age group (≤ 60 years or > 60 years)	0.032	0.776	-0.197	0.261		
Location (converted into dummy variables)	-	0.057	-	-		
Number of participates (> 5000 or ≤ 5000)	0.090	0.421	-0.136	0.315		
Percentage of events (> 10% or ≤ 10 %)	0.099	0.437	-0.159	0.356		
Duration (> median years or ≤ median years)	0.247	0.020	0.043	0.451		
Study quality (NOS score > 7 or ≤ 7)	0.252	0.015	0.054	0.451		
Measurement of weight fluctuation (weight cycle or deviation degree)	-0.120	0.310	-0.359	0.118		
Method for weight ascertainment (self-reported or measured at visit)	0.303	0.005	0.101	0.505		
Follow-up rate (≥ 80% or < 80%)	-0.163	0.141	-0.383	0.057		
Weight change period (≥ 40 years or not)	0.146	0.181	-0.072	0.365		
Duration of assessing weight change (> 10 years or ≤ 10 years)	-0.177	0.109	-0.397	0.043		
Adjustment for physical activity (yes or no)	0.257	0.033	0.023	0.490		
Adjustment for energy intake (yes or no)	0.346	0.003	0.129	0.563		
Adjustment for weight change from baseline (yes or no)	0.086	0.466	-0.153	0.325		

Online Figures

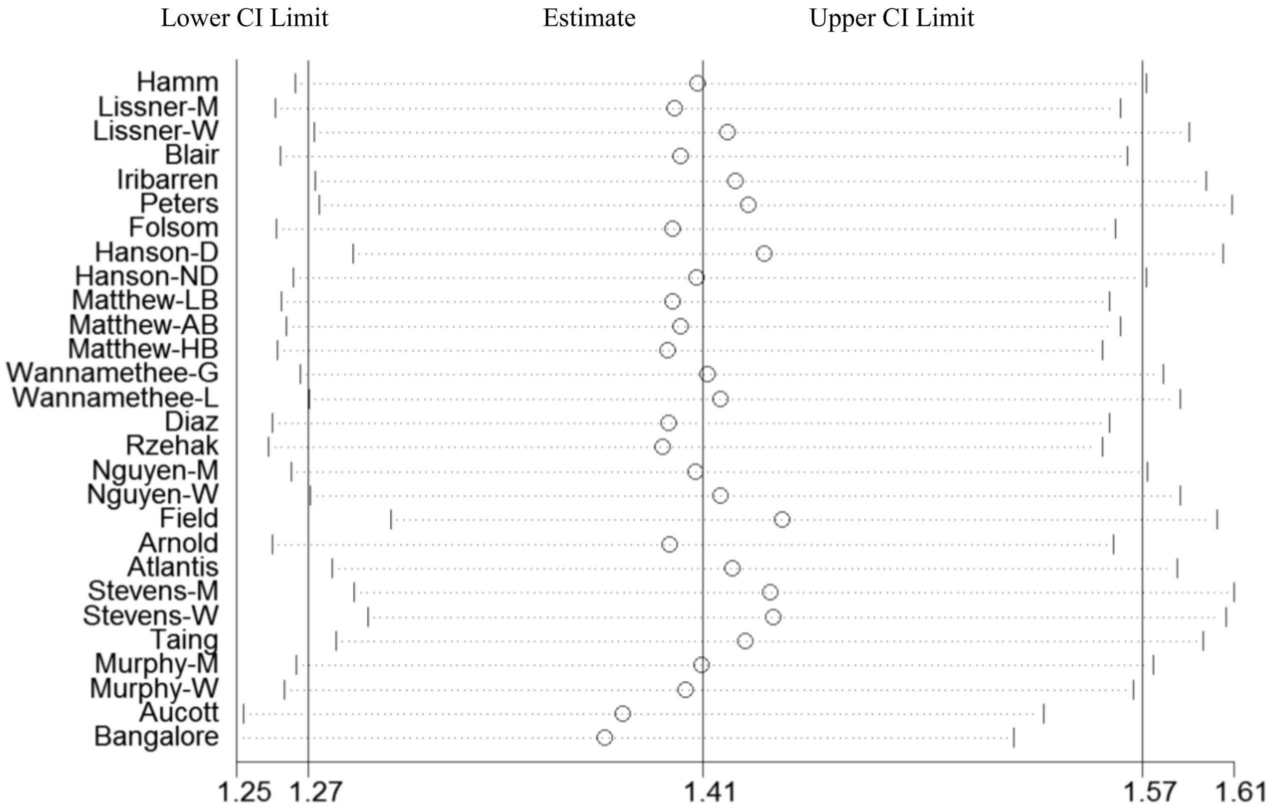

Figure S1. Sensitivity analysis for association of weight fluctuation and risk of all-cause mortality

Estimated RR was calculated by omitting given named study at a time. The summary RR ranged from 1.38 (95% CI 1.25-1.53) when the study by Bangalore et al, 2017 was excluded to 1.44 (95% CI 1.30-1.60) when the study by Field et al, 2009 was excluded.

Abbreviations: CI, confidence interval; RR, relative risk; M, men; W, women; G, weight cycle ending with gain; L, weight cycle ending with loss; D, weight fluctuations in diabetes; ND, weight fluctuations in non-diabetes; LB, weight cycle in low BMI population; AB, weight cycle in average BMI population; HB, weight cycle in high BMI population.

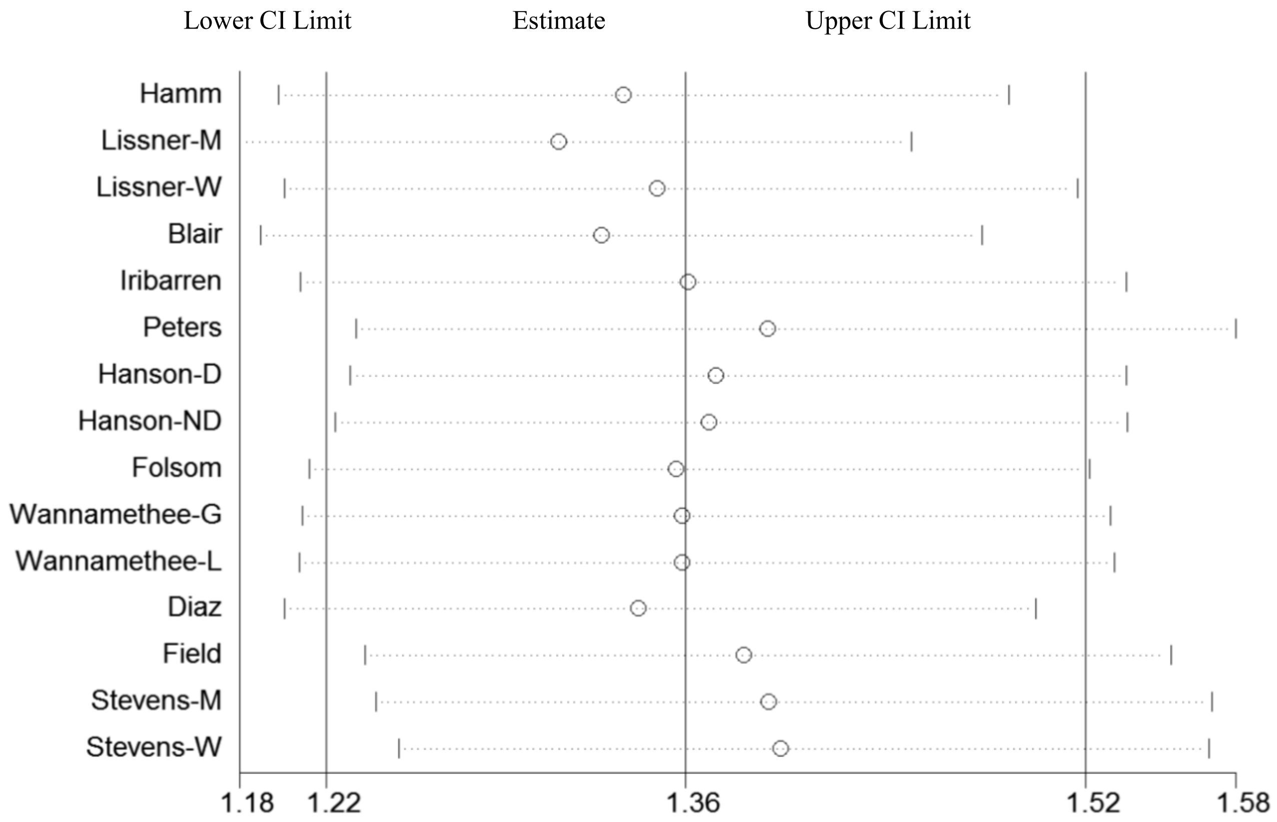

Figure S2. Sensitivity analysis for association of weight fluctuation and risk of CVD mortality

Estimated RR was calculated by omitting given named study at a time. The summary RR ranged from 1.31 (95% CI 1.18-1.45) when the study conducted in men by Lissner et al, 1991 was excluded to 1.40 (95% CI 1.25-1.57) when the study conducted in women by Stevens et al, 2012 was excluded.

Abbreviations: CI, confidence interval; RR, relative risk; M, men; W, women; G, weight cycle ending with gain; L, weight cycle ending with loss; D, weight fluctuations in diabetes; ND, weight fluctuations in non-diabetes.

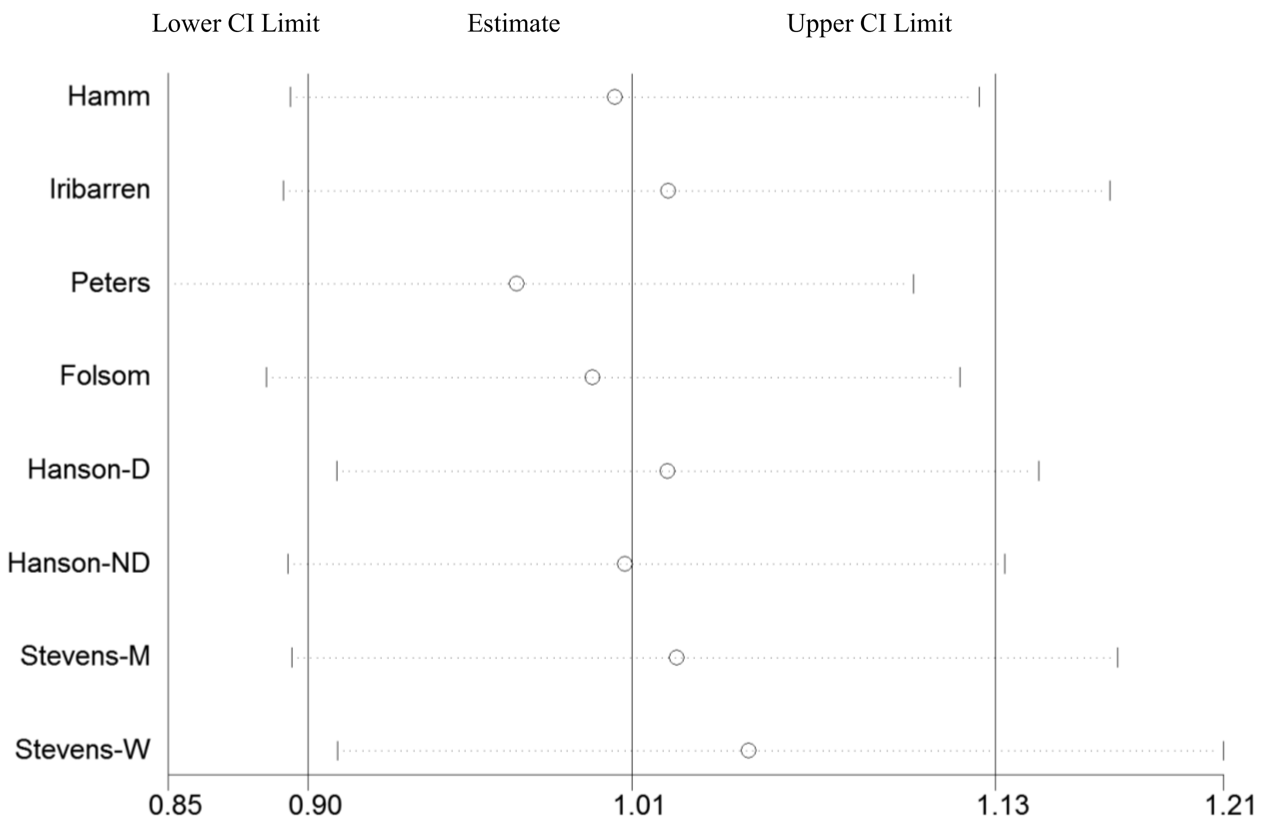

Figure S3. Sensitivity analysis for association of weight fluctuation and risk of cancer mortality

Estimated RR was calculated by omitting given named study at a time. The summary RR ranged from 0.97 (95% CI 0.85-1.10) when the study by Peters et al, 1995 was excluded to 1.02 (95% CI 0.88-1.18) when the study conducted in women by Stevens et al, 2012 was excluded.

Abbreviations: RR, relative risks; CI, confidence interval; M, men; W, women; D, weight fluctuations in diabetes; ND, weight fluctuations in non-diabetes.

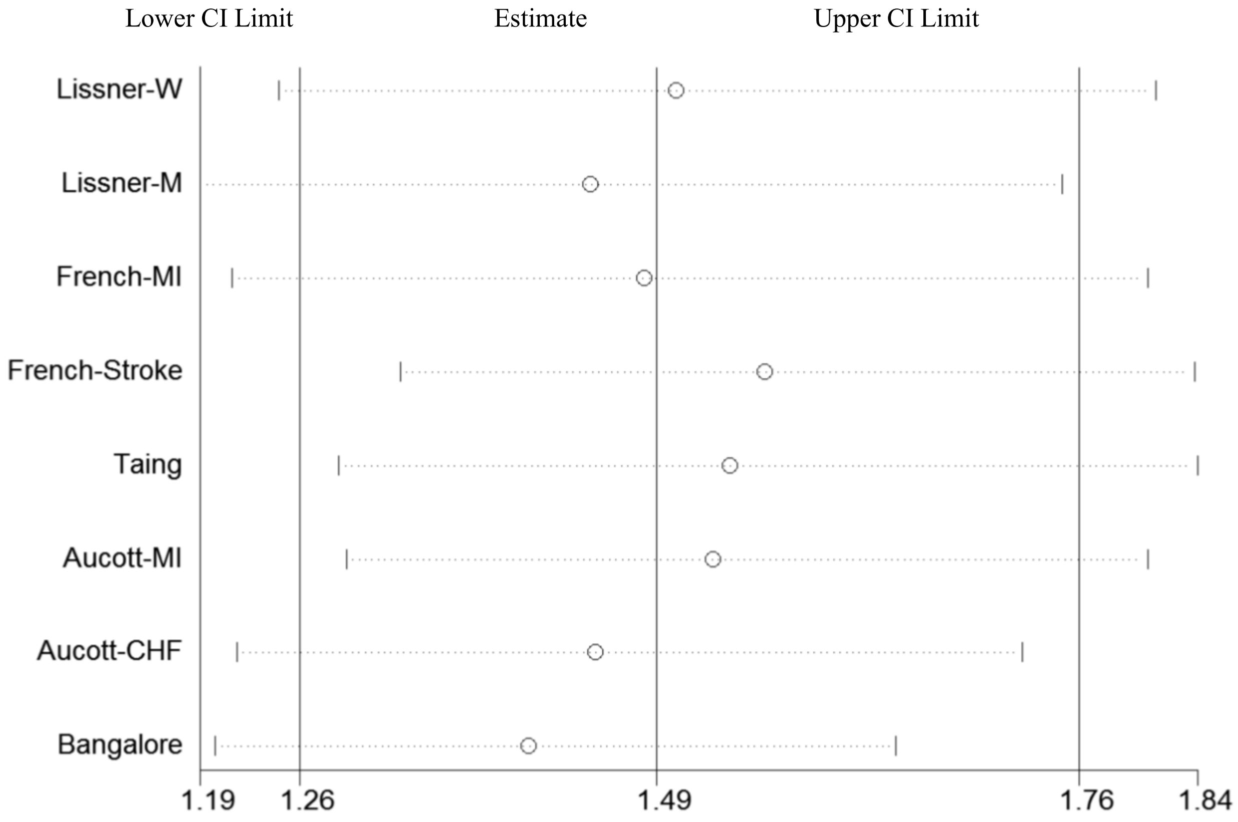

Figure S4. Sensitivity analysis for association of weight fluctuation and risk of CVD

Estimated RR was calculated by omitting given named study at a time. The summary RR ranged from 1.41 (95% CI 1.20-1.64) when the study by Bangalore et al, 2017 was excluded to 1.56 (95% CI 1.32-1.83) when the study by French et al, 1997 was excluded.

Abbreviations: RR, relative risks; CI, confidence interval; CVD, cardiovascular disease; M, men; W, women; MI, myocardial infarction; CHF, congestive heart failure.

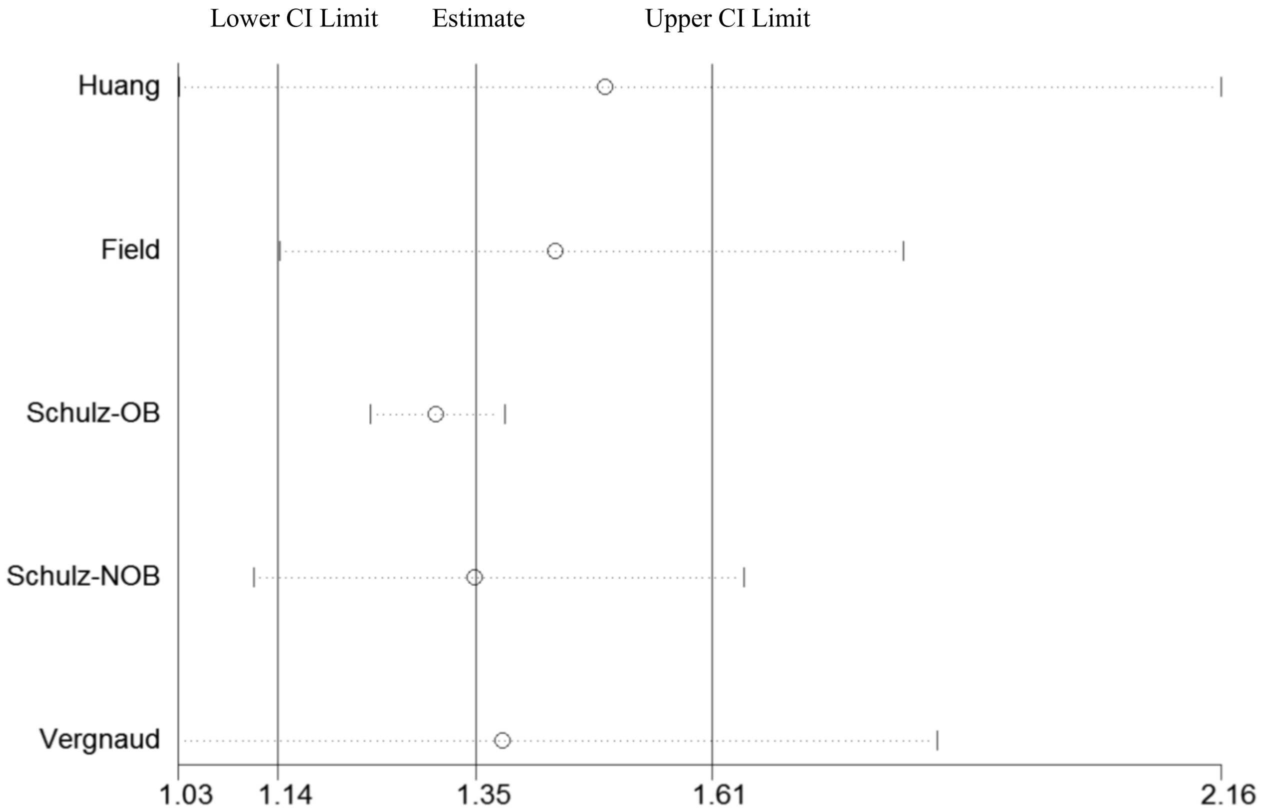

Figure S5. Sensitivity analysis for association of weight fluctuation and risk of hypertension

Estimated RR was calculated by omitting given named study at a time. The summary RR ranged from 1.31 (95% CI 1.24-1.39) when the study conducted in obesity by Sculz et al, 2005 was excluded to 1.49 (95% CI 1.03-2.16) when the study by Huang et al, 1998 was excluded.

Abbreviations: RR, relative risks; CI, confidence interval; NOB, weight fluctuations in non-obese.

References:

1. Hamm P, Shekelle RB, Stamler J. Large fluctuations in body weight during young adulthood and twenty-five-year risk of coronary death in men. *Am J Epidemiol.* 1989;129(2):312-318.

2. Lissner L, Odell PM, D'Agostino RB, et al. Variability of body weight and health outcomes in the Framingham population. *New Engl J Med.* 1991;324(26):1839-1844.

3. Blair SN, Shaten J, Brownell K, Collins G, Lissner L. Body weight change, all-cause mortality, and cause-specific mortality in the Multiple Risk Factor Intervention Trial. *Ann Intern Med.* 1993;119(2):749-757.

4. Iribarren C, Sharp DS, Burchfiel CM, Petrovitch H. Association of weight loss and weight fluctuation with mortality among Japanese American men. *New Engl J Med.* 1995;333(11):686-692.

5. Peters ET, Seidell JC, Menotti A, et al. Changes in body weight in relation to mortality in 6441 European middle-aged men: the Seven Countries Study. *Int J Obesity.* 1995;19(12):862-868.

6. Folsom AR, French SA, Zheng W, Baxter JE, Jeffery RW. Weight variability and mortality: the Iowa Women's Health Study. *Int J Obesity.* 1996;20(8):704.

7. French SA, Folsom AR, Jeffery RW, Zheng W, Mink PJ, Baxter JE. Weight variability and incident disease in older women: the Iowa Women's Health Study. *Int J Obesity.* 1997;21(3):217.

8. Hanson RL, Jacobsson LT, Mccance DR, et al. Weight fluctuation, mortality and vascular disease in Pima Indians. *Int J Obesity.* 1996;20(5):463.

9. Ms MWR, Fredman L, Langenberg P, Magaziner J. Weight, Weight Change, and Mortality in a Random Sample of Older Community-Dwelling Women. *J Am Geriatr Soc.* 1999;47(12):1409-1414.

10. Wannamethee SG, Shaper AG, Walker M. Weight change, weight fluctuation, and mortality. *Archives of Internal Medicine.* 2002;162(22):2575.

11. Diaz VA, Mainous AG, Everett CJ. The association between weight fluctuation and mortality: results from a population-based cohort study. *Journal of Community Health.* 2005;30(3):153-165.

12. Nguyen ND, Center JR, Eisman JA, Nguyen TV. Bone loss, weight loss, and weight fluctuation predict mortality risk in elderly men and women. *Journal of Bone & Mineral Research.* 2007;22(8):1147-1154.

13. Rzehak P, Meisinger C, Woelke G, Brasche S, Strube G, Heinrich J. Weight change, weight cycling and mortality in the ERFORT Male Cohort Study. *Eur J Epidemiol.* 2007;22(10):665-673.

14. Huang Z, Willett WC, Manson JE, et al. Body weight, weight change, and risk for hypertension in women. *Ann Intern Med.* 1998;128(2):81-88.

15. Field AE, Byers T, Hunter DJ, et al. Weight Cycling, Weight Gain, and Risk of Hypertension in Women. *Am J Epidemiol.* 1999;150(6):573-579.

16. Field AE, Malspeis S, Willett WC. Weight cycling and mortality among middle-aged or older women. *Archives of Internal Medicine.* 2009;169(9):881.

17. Arnold AM, Newman AB, Cushman M, Ding J, Kritchevsky S. Body Weight Dynamics and Their Association With Physical Function and Mortality in Older Adults: The Cardiovascular Health Study. *Journals of Gerontology.* 2010;65(1):63.

18. Atlantis E, Browning C, Kendig H. Body mass index and unintentional weight change associated with all-cause mortality in older Australians: the Melbourne Longitudinal Studies on Healthy Ageing (MELSHA). *Age & Ageing.* 2010;39(5):643-646.

19. Taing KY, Ardern CI, Kuk JL. Effect of the Timing of Weight Cycling During Adulthood on Mortality Risk in Overweight and Obese Postmenopausal Women. *Obesity.* 2012;20(2):407-413.

20. Stevens VL, Jacobs EJ, Sun J, et al. Weight cycling and mortality in a large prospective US study. *Am J Epidemiol.* 2012;175(8):785.

21. Murphy RA, Patel KV, Kritchevsky SB, et al. Weight change, body composition, and risk of mobility

disability and mortality in older adults: a population-based cohort study. *J Am Geriatr Soc.* 2014;62(8):1476-1483.

22. Aucott LS, Philip S, Avenell A, Afolabi E, Sattar N, Wild S. Patterns of weight change after the diagnosis of type 2 diabetes in Scotland and their relationship with glycaemic control, mortality and cardiovascular outcomes: a retrospective cohort study. *Bmj Open.* 2016;6(7):e10836.

23. Bangalore S, Fayyad R, Laskey R, DeMicco DA, Messerli FH, Waters DD. Body-Weight Fluctuations and Outcomes in Coronary Disease. *New Engl J Med.* 2017.

24. Schulz M, Liese AD, Boeing H, Cunningham JE, Moore CG, Kroke A. Associations of short-term weight changes and weight cycling with incidence of essential hypertension in the EPIC-Potsdam Study. *J Hum Hypertens.* 2005;19(1):61-67.

26. Vergnaud AC, Bertrais S, Oppert JM, et al. Weight fluctuations and risk for metabolic syndrome in an adult cohort. *Int J Obesity.* 2007;32(2):315.
