# Supplementary material for: Body-Weight Fluctuation Was Associated With Increased Risk for Cardiovascular Disease, All-Cause and Cardiovascular Mortality: A Systematic Review and Meta-Analysis
Source: Front Endocrinol (Lausanne). 2019 Nov 8;10:728. doi: 10.3389/fendo.2019.00728 (PMC6856014; doi:10.3389/fendo.2019.00728)
Supplement: Supplementary file 2 [file Table_1.pdf]

Table 1. Descriptive characteristics of 25 included articles

Author	Year	Participants	Number of participants (Sex)	Design, length of follow-up	Definition for weight fluctuation	Weight fluctuation category and RR (95% CI)	Covariates in fully adjusted model
Hamm, et al (16)	1989	Men who had been employed the Western Electric Company's Hawthorne Works in Chicago.	1,959 (Male)	Cohort, 25 years	Weight-change was divided into four groups: gain only group (maximum gain during any one five-year period was 10% or more), no change group (largest gain or largest loss less than 5% and weight at initial examination differed by less than 5 % from the reported weight at age 20), gain and loss group (maximum gain during any one 5-year period and maximum loss during another 5-year period were each 10% or more), all others group (remain patients).	All-cause mortality: no change, 1.00 (reference); gain only, 1.40 (1.00-2.10); gain and loss 1.5 (1.00-2.30); all others, 1.30 (1.00-1.70). CHD mortality: no change 1.00 (reference); gain only, 1.10 (0.60-2.00); gain and loss 2.00 (1.20-3.50); all others, 1.2 (0.80-1.90). Cancer mortality: no change 1.00 (reference); gain only, 3.00 (1.40-6.50); gain and loss 1.50 (0.60-4.10); all others, 1.90 (1.00-3.60).	Age, serum cholesterol, systolic blood pressure, cigarette smoking, intake of ethanol and BMI, intake of nutrients and physical activity.
Lissner, et al (8)	1991	Male and female residents of Framingham, Massachusetts, who were initially free of CHD.	3,130 (Male and female)	Cohort, 32 years	CV for each subject's BMI was calculated at nine times points from the age of 25 (recalled weight) through 8th examination. Then subjects were into tertiles of CV of body-weight.	Men:<br>All-cause mortality: tertile 1, 1.00 (reference); tertile 3, 1.65 (1.32-2.06).<br>Morbidity due to CHD: tertile 1, 1.00 (reference); tertile 3, 1.78 (1.32-2.39).<br>Mortality from CHD: tertile 1, 1.00 (reference); tertile 3, 1.93 (1.35-2.77).<br>Women: All-cause mortality: tertile 1, 1.00 (reference); tertile 3, 1.27 (1.01-1.61).<br>Morbidity due to CHD: tertile 1, 1.00 (reference); tertile 3, 1.38	Age, BMI, slop of BMI and BMI over the same period, smoking statues, physical activity, serum cholesterol concentration, results of glucose-tolerance tests, and systolic blood pressure.

Table 1. Descriptive characteristics of 25 included articles (continued).

Author	Year	Participants	Number of participants (Sex)	Design, length of follow-up	Definition for weight fluctuation	Weight fluctuation category and RR (95% CI)	Covariates in fully adjusted model
						(0.98-1.94).<br>Mortality from CHD: tertile 1, 1.00 (reference); tertile 3, 1.55 (1.09-2.21).	
Blair, et al (23)	1993	Men who were in the upper 10% to 15% of risk for coronary heart disease.	10,529 (Male)	Cohort. 3.8 years	ISD was calculated from measured weights obtained at clinic visits during a 6- to 7-year period.	Quartile of ISD:<br>All-cause mortality: quartile 1, 1.00 (reference); quartile 2, 1.04 (0.77-1.41); quartile 3, 1.46 (1.09-1.96), quartile 4, 1.64 (1.21-2.23).<br>CVD mortality: quartile 1, 1.00 (reference); quartile 2, 1.15 (0.77-1.70); quartile 3, 1.47 (0.99-2.16), quartile 4, 1.85 (1.25-2.75).	Age, race, baseline values of diastolic blood pressure, diuretic use, serum cholesterol, BMI, number of cigarettes smoked per day, reported number of alcoholic drinks consumed per week, and initial level of physical activity compared with the peer group.

Table 1. Descriptive characteristics of 25 included articles (continued).

Author	Year	Participants	Number of participants (Sex)	Design, length of follow-up	Definition for weight fluctuation	Weight fluctuation category and RR (95% CI)	Covariates in fully adjusted model
Iribarren, et al (5)	1995	Japanese American men who were living on the island of Oahu, Hawaii. No a priori exclusions were made because of pre-existing medical conditions.	6,573 (Male)	Cohort, 14.5 years	RMSE around the linear slope of weight was calculated as $(\sum di^2/ (n-p-1))$ 1/2 for each subject based on measurement of each visit and then participants were into quintiles of measures of body-weight variability.	All-cause mortality: quintile 1, 1.00 (reference); quintile 2, 1.14 (0.95-1.37); quintile 3, 1.07 (0.89-1.29); quintile 4, 1.01 (0.84-1.21); quintile 5, 1.25 (1.05-1.48).<br>CVD mortality: quintile 1, 1.00 (reference); quintile 2, 0.99 (0.69-1.42); quintile 3, 1.08 (0.77-1.52); quintile 4, 1.11 (0.79-1.55); quintile 5, 1.41 (1.03-1.93).<br>Cancer mortality: quintile 1, 1.00 (reference); quintile 2, 1.10 (0.83-1.46); quintile 3, 0.96 (0.72-1.27); quintile 4, 0.91 (0.69-1.21); quintile 5, 0.97 (0.73-1.27).	Age, average weight, slope of weight, smoking status, and number of cigarettes smoked per day, base-line alcohol consumption, level of physical activity, total caloric intake, job and pre-existing disease.
Peters, et al (24)	1995	Men who were from seven countries and without diabetes or cancer.	6,441 (Male)	Perspective follow-up study, 5 years.	Participants were divided in four groups based on the 1st and 3rd examination: constant weight (weight loss or gain within 2 kg); weight gain (weight gain more than 2 kg); weight loss (weight loss more than 2 kg); and weight fluctuating (weight gain more than 2 kg heavier than the highest weight at examination 1 or 3). Weight variability was also expressed by CV mean and CVreg.	Quartile of CVreg:<br>All-cause mortality: quartile 1, 1.00 (reference); quartile 2, 0.98 (0.86-1.12); quartile 3, 1.01 (0.89-1.15); quartile 4, 1.21 (1.07-1.38).<br>CVD mortality: quartile 1, 1.00 (reference); quartile 2, 0.92 (0.77-1.11); quartile 3, 1.04 (0.87-1.25); quartile 4, 1.21 (1.02-1.45).<br>Cancer mortality: quartile 1, 1.00 (reference); quartile 2, 1.07 (0.84-1.38); quartile 3, 0.97 (0.76-1.25); quartile 4, 1.17 (0.91-1.50).	Age, geographical region, body weight at 3rd examination, smoking pattern and slope of the regression line.

Table 1. Descriptive characteristics of 25 included articles (continued).

Author	Year	Participants	Number of participants (Sex)	Design, length of follow-up	Definition for weight fluctuation	Weight fluctuation category and RR (95% CI)	Covariates in fully adjusted model
Folsom, et al (13)	1996	Iowan women who were free of cancer and heart disease.	33,760 (Female)	Cohort, 6 years	Weight variability consisted of categories of body weight change: a large cycle: gained ≥ 10% of body weight during one interval and lost ≥ 10% during another interval; a small cycle category: gained ≥ 5% but < 10% during one interval, and lost between 5 and 10% during another interval; no change category: differed by < 5% from their reported weight at age 18 years, and whose weight change during any interval was < 5%.	All-cause mortality: no change, 1.00 (reference); small cycles, 1.42 (0.90-2.30); large cycles, 1.81 (1.20-2.80).<br>CVD mortality: no change, 1.00 (reference); small cycles, 1.4 (0.60-3.20); large cycles, 1.68 (0.80-3.60).<br>Cancer mortality: no change, 1.00 (reference); small cycles, 0.99 (0.50-1.90); large cycles, 1.38 (0.80-2.50).	Age, waist-hip ratio, BMI, square of BMI, the regression slope of weight, smoking status, pack years of cigarettes, education, physical activity, alcohol, and hormone replacement.
French, et al (25)	1997	Iowan women who were free of cancer and heart disease.	33,834 (Female)	Cohort, 6 years	RMSE around the linear slope of weight in relation to age was calculated for each subject.	Quartiles of RMSE:<br>MI: quartile 1, 1.00 (reference); quartile 2, 1.11 (0.85-1.44); quartile 3, 1.08 (0.83-1.40); quartile 4, 1.51 (1.16-1.95).<br>Stroke: quartile 1, 1.00 (reference); quartile 2, 0.91 (0.69-1.21); quartile 3, 1.04 (0.79-1.37); quartile 4, 1.14 (0.86-1.51).	Age, baseline BMI, square of BMI, slope of weight on age, waist-hip ratio, smoking status, pack years of cigarettes, education, physical activity, marital status, hormone replacement, hypertension and diabetes (in MI and stroke).
Hanson, et al (28)	1996	Gila River Indian Community residents.	1,338 (Male and female)	A longitudinal study, 9.3 years	RMSE of regression line of weight was used as index of weight fluctuation. Then subjects were divided into 2 groups by the median value of RMSE in each group (2.8 kg for diabetes; 3.2 kg for non-diabetic subjects).	RR of high RMSE compared with low RMSE<br>In patients with diabetes:<br>All-cause mortality: 1.00 (0.80-1.30).<br>CVD mortality: 0.90 (0.40-1.70).<br>Cancer mortality: 0.50 (0.20-1.20).	Age, sex, BMI, slope of body weight on age, nephropathy, insulin treatment, smoking and the time and rate of weight gain between initial and baseline examinations.

Table 1. Descriptive characteristics of 25 included articles (continued).

Author	Year	Participants	Number of participants (Sex)	Design, length of follow-up	Definition for weight fluctuation	Weight fluctuation category and RR (95% CI)	Covariates in fully adjusted model
						In non-diabetes:<br>All-cause mortality: 1.50 (1.00-2.10).<br>CVD mortality: 1.10 (0.50-2.30)<br>Cancer mortality: 1.30 (0.50-3.50)	
Reynolds, et al (12)	1999	Older, community-dwelling, white women. Forty percent of the respondents had a pre-existing illness.	648 (Female)	Cohort, 6 years	Participants were divided into four groups: weight gain (more than a 4.5% weight gain between the first and second interview, more than a 4.5% gain between the second and third interview, or more than a cumulative 7.5% gain between the first and third interview), weight loss (similarly to weight gain, but using percentage loss), weight cycling (those who had more than a 4.5% gain or loss in BMI between the first and second interview, and more than a 4.5% change in BMI in the opposite direction between the 2nd and 3rd interviews), and no change (not belong to any of the previous three categories).	All-cause mortality:<br>BMI*Weight Change Subgroups:<br>Average* gain No Change: 1.00 (reference); low* cycle: 2.64 (1.1 0-6.38); high* cycle: 2.94 (1.21-7.15); average*cycle: 2.24 (0.94-5.35).	Age, education, smoking, alcohol usage, an interaction between weight change and baseline BMI, and pre-existing illness, including cancer, heart trouble, stroke, and diabetes.
Wanname-thee, et al (11)	2002	Men selected from the age-sex registers of one general practice in each of 24 towns	5,608 (Male)	Perspective study, 8 years	Participants were divided into 5 groups: stable (less than 4% change in body weight); sustained gain (weight gain≥ 4% and without weight loss ≥ 4% during follow-up;	All-cause mortality: stable 1.00 (reference); loss-gain, 1.40 (1.06-1.85); gain-loss, 1.31 (1.02-1.68).<br>CVD mortality: stable 1.00 (reference); loss-gain, 1.45 (0.98-	Age, social class, smoking status, physical activity, initial BMI, pre-existing CVD, diabetes mellitus, cancer and poor health.

Table 1. Descriptive characteristics of 25 included articles (continued).

Author	Year	Participants	Number of participants (Sex)	Design, length of follow-up	Definition for weight fluctuation	Weight fluctuation category and RR (95% CI)	Covariates in fully adjusted model
		in England, Wales, and Scotland.			sustained loss (weight loss ≥ 4% and without regain); loss-gain (weight loss in the first 5 years followed by weight gain in the last years during follow-up); gain-loss (weight gain in the first 5 years followed by weight loss in the last years during follow-up).	2.15); gain-loss, 1.44 (1.02-2.04).	
Diaz, et al (20)	2005	Non-institutionalized civilian United States population.	8,479 (Male and female)	Cohort, 21 years	Weight gain was defined as increase of ≥ 3.0 BMI units from initial to final BMI. Weight loss was defined as decrease of ≥ 3.0 units from initial to final BMI. Weight fluctuation was defined as a sum of deviations > 5.04 for those with < 3.0 units difference from the initial to final BMI.	All-cause mortality: stable weight-non-obese, 1.00 (reference); stable weight-obese 1.35 (0.64-2.85); weight gain 1.10 (0.72-1.67); weight loss 3.36 (2.47-4.55); weight fluctuation 1.83 (1.25-2.69). CVD mortality: stable weight, non-obese 1.00 (reference); stable weight, obese 2.37 (0.97-5.75); weight gain 1.24 (0.62-2.49); weight loss 4.22 (2.60-6.84); weight fluctuation 1.86 (1.10-3.15).	Age, gender, race, initial BMI, smoking status and charlson comorbidity index, which was specifically designed to control for health status in longitudinal studies with mortality as an outcome by accounting for age, cardiovascular risk factors and other comorbid diseases.
Nguyen, et al (19)	2007	Participants of white background who participated in the Dubbo Osteoporosis Epidemiology Study.	1,703 (Male and female)	Cohort, 13 years	Categorical variables of weight change derived from the PC scores (PC1: normal weight, overweight, obese; PC2: weight loss, stable weight, weight gain; PC3 and PC4: non-cycling, weight cycling) were used to estimate associations between BMI patterns and the hazard of type	Men:<br>All-cause mortality: weight fluctuation <3%, 1.00 (reference); weight fluctuation ≥ 3%, 1.50 (1.10-2.00).<br>Women:<br>All-cause mortality: weight fluctuation <3%, 1.00 (reference);	Baseline BMD, age, smoking status, weight change, and concomitant diseases, including CVD, all types of cancer, and diabetes mellitus.

Table 1. Descriptive characteristics of 25 included articles (continued).

Author	Year	Participants	Number of participants (Sex)	Design, length of follow-up	Definition for weight fluctuation	Weight fluctuation category and RR (95% CI)	Covariates in fully adjusted model
					2 diabetes.	weight fluctuation ≥ 3%, 1.30 (1.00-1.70).	
Rzehak, et al (18)	2007	Men drawn by a random sample of the population born 1915–1934 in the city of Erfurt.	1,160 (Male)	Cohort, 15 years	Weight change categories: stable weight-non-obese, stable weight-obese, weight gain, weight loss, weight fluctuations. Weight gain and weight loss was defined as an increase, respective decrease of ≥ 3 BMI units from initial to final BMI measurements in the first 15 years of observation. Weight fluctuations were ascribed to those respondents, whose initial and final BMI differed by < 3 kg/m^2 and whose sum of absolute deviations were > 3.49 BMI units.	All-cause mortality: stable non-obese: 1.00 (reference); stable obese: 1.16 (0.69–1.94); gain: 1.15 (0.70–1.88); loss: 1.81 (0.99–3.31); fluctuations: 1.86 (1.31-2.66).	Age, smoking status, and pre-existing disease, including MI, angina pectoris, ischemic heart disease, stroke, diabetes mellitus or hypertension.
Huang, et al (17)	1998	U.S. female nurses aged 30 to 55 years.	82,473 (Female)	Cohort, 16 years	Women were divided into nine groups: weight loss of 10 kg or more, loss of 5.0 to 9.9 kg, loss of 2.1 to 4.9 kg, loss or gain of 2.0 kg or less, gain of 2.1 to 4.9 kg, gain of 5.0 to 9.9 kg, gain of 10.0 to 19.9 kg, gain of 20.0 to 24.9 kg, and gain of 25 kg or more. The stable weight group (loss or gain ≤ 2.0 kg) was the referent. Otherwise, they were classified into an unstable weight group.	Hypertension: Loss or gain ≤ 2 kg: 1.00 (reference); unstable weight: 1.31 (1.24-1.39)	Age, BMI at age 18 years, height, family history of MI, parity, oral contraceptive use, menopausal status and postmenopausal use of hormone and smoking status.
Field, et al	1999	Female nurses, 25	46,224	Cohort, 6	Weight change categories: stable	All-cause mortality: stable non-	Age, smoking status, and

Table 1. Descriptive characteristics of 25 included articles (continued).

Author	Year	Participants	Number of participants (Sex)	Design, length of follow-up	Definition for weight fluctuation	Weight fluctuation category and RR (95% CI)	Covariates in fully adjusted model
(21)		to 43 years of age at entry	(Female)	years	weight-non-obese, stable weight-obese, weight gain, weight loss, weight fluctuations. Weight gain and weight loss was defined as an increase, respective decrease of ≥ 3 BMI units from initial to final BMI measurements in the first 15 years of observation. Weight fluctuations were ascribed to those respondents, whose initial and final BMI differed by < 3 kg/m^2 and whose sum of absolute deviations were > 3.49 BMI units.	obese: 1.00 (reference); stable obese: 1.16 (0.69–1.94); gain: 1.15 (0.70–1.88); loss: 1.81 (0.99–3.31); fluctuations: 1.86 (1.31-2.66).	pre-existing disease, including MI, angina pectoris, ischemic heart disease, stroke, diabetes mellitus or hypertension.
Field, et al (6)	2009	Middle-aged or older women in the Nurses' Health Study and had no history of cancer (other than nonmelanoma skin cancer) or heart disease.	44,882 (Female)	Cohort, 12 years	Women who reported they had intentionally lost ≥ 20 lbs (9 kg) ≥ 3 times were classified as severe weight cyclers. Women who had intentionally lost >10 lbs (4.5 kg) ≥ 3 times, but who did not meet the criteria for severe weight cycling, were classified as mild weight cyclers. Women who had intentionally weight loss, but did not meet the criteria for mild weight cyclers were classified as non-cycler.	Hypertension:<br>Non-cycler, 1.00 (reference); mild cycler, 1.15 (1.00-1.33); severe cycle, 1.13 (0.79-1.61).	Age, BMI at baseline, activity level, smoking status, and alcohol intake.
Arnold, et al (14)	2010	Participants were recruited from random samples of	3,278 (Male and female)	Cohort, 7 years	Participants were classified as stable, if weight changed by less than 5% from the prior year and from	All-cause mortality: stable: 1.00 (reference); cycling (unstable): 1.66 (1.38, 2.00).	Age, sex, height, race, high school education, current smoking, diabetes,

Table 1. Descriptive characteristics of 25 included articles (continued).

Author	Year	Participants	Number of participants (Sex)	Design, length of follow-up	Definition for weight fluctuation	Weight fluctuation category and RR (95% CI)	Covariates in fully adjusted model
		Medicare eligibility lists in four communities。			baseline; weight loss (gain) if the participant lost (gained) 5% of body weight between consecutive visits or from baseline, without an intervening 5% gain (loss); and unstable or cycling after undergoing both a 5% loss and gain.		osteoporosis, self-reported health, hypertension, lung ailments, cancer, congestive heart failure, coronary heart disease, claudication, stroke, or transient ischemic attack.
Atlantis, et al (10)	2010	All residents of private dwellings in metropolitan Melbourne, aged 65+ years in 1994.	1,000 (Male and female)	Prospective cohort, 12 years	Study participants were grouped into the following four categories: (1) no change (at baseline and all follow-ups), (2) gain (reported at least once), (3) loss (reported at least once) and (4) fluctuation (gain as well as loss reported at least once at two different time points).	All-cause mortality: none: 1.00 (reference); gain 5 kg: 0.78 (0.46-1.32); loss 5 kg: 0.46 (0.32-0.66); fluctuation: 0.72 (0.29-1.76)	Final multivariate model retains only significant demographic, lifestyle, functional health and chronic disease covariates.
Taing, et al (15)	2012	Women with BMI≥ 25 and without baseline disease (CVD, cancer, diabetes).	47,473 (Female)	Cohort, 7 years	Weight change patterns was categorized into stable (weight changes of < 5% during all 3 time-periods), weight gain (weight-gain ≥ 5% at least one period, but without a period of weight-loss ≥ 5%), weight loss (weight-loss ≥ 5% at least one period, but without a period of weight-gain ≥ 5%), weight cycling (weight-gain and loss ≥ 5%).	All-cause mortality: stable: 1.00 (reference); weight cycling: 1.08 (0.79-1.48).	BMI, age, ethnicity, income, education, alcohol intake, hormone replacement therapy, smoking status, and physical activity and pre-existing diseases (CVD, diabetes, and cancer).
Stevens, et al (7)	2012	Participants in the Nutrition Cohort of the CPS-II who	122,638 (Male and female)	Cohort, 16 years	A weight cycle was defined as an intentional loss of 10 or more pounds (4.5 kg) followed by regain of that	Men:<br>All-cause mortality: quintile 1, 1.00 (reference); quintile 2, 0.93 (0.89-	Age, alcohol consumption, race, smoking status, educational level, physical

Table 1. Descriptive characteristics of 25 included articles (continued).

Author	Year	Participants	Number of participants (Sex)	Design, length of follow-up	Definition for weight fluctuation	Weight fluctuation category and RR (95% CI)	Covariates in fully adjusted model
		were free of cancer, a history of heart attack, stroke, emphysema, or other lung diseases in 1992			weight based on 2 questions about intentional weight loss and regain on the 1992 baseline questionnaire. Then the weight cycles were divided into 5 categories by the number of weight cycles: quintile 1 (0), quintile 2 (1-4 cycles), quintile 3 (5-9 cycles), quintile 4 (10-19 cycles), and quintile 5 (≥ 20 cycles).	0.97); quintile 3, 0.88 (0.81-0.94); quintile 4, 0.96 (0.88-1.05); quintile 5, 1.03 (0.89-1.19).<br>CVD mortality: quintile 1, 1.00 (reference); quintile 2, 0.93 (0.87-0.99); quintile 3, 0.93 (0.82-1.05); quintile 4, 0.97 (0.83-1.13); quintile 5, 1.14 (0.91-1.44).<br>Cancer mortality: quintile 1, 1.00 (reference); quintile 2, 0.97 (0.90-1.03); quintile 3, 0.83 (0.72-0.95); quintile 4, 0.93 (0.79-1.09); quintile 5, 0.96 (0.74-1.26).<br>Women:<br>All-cause mortality: quintile 1, 1.00 (reference); quintile 2, 0.93 (0.89-0.98); quintile 3, 0.95 (0.88-1.02); quintile 4, 0.93 (0.85 -1.01); quintile 5, 0.99 (0.88-1.12).<br>CVD mortality: quintile 1, 1.00 (reference); quintile 2, 0.91 (0.83-0.99); quintile 3, 0.91 (0.79-1.04); quintile 4, 0.93 (0.80- 1.09); quintile 5, 1.11 (0.91-1.36).<br>Cancer mortality: quintile 1, 1.00 (reference); quintile 2, 0.93 (0.86-1.01); quintile 3, 0.94 (0.83-1.07); quintile 4, 0.89 (0.77-1.03); quintile	activity level, BMI in 1982, weight change from 18 years of age to 1982, history of high blood pressure, history of diabetes, and total energy intake.

Table 1. Descriptive characteristics of 25 included articles (continued).

Author	Year	Participants	Number of participants (Sex)	Design, length of follow-up	Definition for weight fluctuation	Weight fluctuation category and RR (95% CI)	Covariates in fully adjusted model
						5, 0.94 (0.78-1.15).	
Murphy, et al (27)	2014	Participants were recruited from a random sample of white Medicare beneficiaries and all black Medicare-eligible residents and were without pre-existing disease.	1,975 (Male and female)	Cohort, 8 years	Weight was categorized as stable, loss, gain, or cycling (gain and loss).	Men:<br>All-cause mortality: weight sable: 1.00 (reference); weight cycling: 1.45 (1.04-2.03).<br>Women:<br>All-cause mortality: weight sable: 1.00 (reference); weight cycling: 1.61 (1.14-2.28)	Age, race, education, study site, BMI, smoking status, physical activity, depressive symptoms, cancer, diabetes mellitus, hip fracture, hypertension, MI, stroke, incidence of hospitalization, and days of hospitalization, year 6 lean mass, year 6 fat mass, percentage change in lean mass and percentage change in fat mass.
Aucott, et al (26)	2016	Overweight/obese patients with incident diabetes diagnosed between 2002 and 2006.	29,316 (Male and female)	Retrospective cohort, 5.2 years	Weight variability was categorized into 4 groups by CV of weight change: quartile 1 (CV < 2.5%), quartile 2 (2.5% to < 5%), quartile 3 (5% to <10%), quartile 4 (≥ 10%).	All-cause mortality: quartile 1, 1.00 (reference), quartile 2, 1.14 (0.94-1.38); quartile 3, 1.77 (1.41-2.23); quartile 4, 2.49 (1.68-3.68).<br>MI: quartile 1, 1.00 (reference), quartile 2, 1.15 (0.94-1.41); quartile 3, 1.57 (1.21-2.03); quartile 4, 0.99 (0.54-1.84).<br>CHF: quartile 1, 1.00 (reference), quartile 2, 1.68 (1.30-2.15); quartile 3, 2.00 (1.46- 2.75); quartile 4, 2.23 (1.26-3.96).	Age, BMI, sex, smoking status, deprivation, HbA1c control at 2 years, and antidiabetic medication regimes.
Bangalore, et al (9)	2017	Patients with clinically evident coronary artery	9,505 (Male and female)	Post hoc analysis, 4.7 years	Average successive variability, which was defined as the average absolute difference between successive values,	All-cause mortality: quintile 1, 1.00 (reference); quintile 5, 2.24 (1.74-2.89).	Age, sex, race, diabetes, hypertension, and smoking; mean weight and weight

Table 1. Descriptive characteristics of 25 included articles (continued).

Author	Year	Participants	Number of participants (Sex)	Design, length of follow-up	Definition for weight fluctuation	Weight fluctuation category and RR (95% CI)	Covariates in fully adjusted model
		disease and levels of low-density lipoprotein (LDL) cholesterol below 130 mg per deciliter (3.4 mmol per liter) who had been randomly assigned to receive either 10 mg or 80 mg of atorvastatin per day.			SD, CV, and variability independent of the mean was used as measurement of body-weight variability and patients were divided into quintiles of measures of body-weight variability.	Any cardiovascular event: quintile 1, 1.00 (reference); quintile 5, 1.85 (1.62-2.11).	change; treatment; baseline levels of LDL cholesterol, HDL cholesterol, total cholesterol, and triglycerides; chronic kidney disease and chronic heart failure; and time between initial and final weight measurements.
Schulz, et al (29)	2005	Caucasian men and women were recruited into the study at the recruitment examination (baseline) between 1994 and 1998.	12,362 (Male and female)	Cohort, 2 years	Subjects were assigned to one of the following groups: weight gain only (if they experienced weight gain but no weight loss) with further separation into intentional and unintentional weight gain, weight loss only (if they experienced short-term weight loss but no short-term weight gain) with further separation into intentional and unintentional weight loss, weight cycling (if they experienced unintentional weight gain and intentional weight loss), other cycling not meeting the above-mentioned criteria for weight cycling, and stable weight, if no short-term	Hypertension:<br>Obese: weight stable: 1.00 (reference); weight cycling: 4.16 (1.48-11.7).<br>Non-obese: weight stable: 1.00 (reference); weight cycling: 1.80 (0.59-5.54).	Age, gender, education, smoking, alcohol intake, leisure-time physical activity, baseline systolic and diastolic blood pressure.

Table 1. Descriptive characteristics of 25 included articles (continued).

Author	Year	Participants	Number of participants (Sex)	Design, length of follow-up	Definition for weight fluctuation	Weight fluctuation category and RR (95% CI)	Covariates in fully adjusted model
					weight change was reported (referent category).		
Vergnaud, et al (22)	2007	Participants of the SU.VI.MAX study who were free of cancer or CVD.	3,553 (Male and female)	Cohort, 7.5 years	Weight fluctuation was defined as at least two weight variations in opposite directions. Subjects with weight fluctuation were divided into 4 groups (tertile 0-3).	Hypertension: tertile 1, 1.00 (reference); tertile 2, 1.09 (0.82-1.44), tertile 3 1.43 (1.08-1.89).	Age, sex, smoking status, education level and physical activity and relative weight change.

Abbreviations: BMI, body mass index; BMD, bone mineral density; CHD, coronary heart disease; CHF, congestive heart failure; CV, coefficient of variation; CV mean, coefficient of variation around the mean; CVreg, coefficient of variation around the regression line of three body weights, which was computed as 100 times the square root of the mean square error divided by the mean weight; CVD, cardiovascular diseases; LDL, low density lipoprotein; MI, myocardial infarction; RMSE, root mean square error; SD, standard deviation; ISD, intrapersonal standard deviation of weight.
